# Polystyrene microplastics impair sheep spermatogenesis by disrupting rumen microbiota-derived butyrate signaling

**DOI:** 10.1186/s40104-026-01490-z

**Published:** 2026-09-09

**Authors:** Juanjuan Song, Keyan Ma, Xingcai Qi, Dengpan Li, Xingxu Zhao, Youji Ma

**Affiliations:** 1 College of Animal Science and Technology, Gansu Agricultural University, Lanzhou, 730070 China; 2 Gansu Key Laboratory of Animal Generational Physiology and Reproductive Regulation, Lanzhou, 730070 China

**Keywords:** Butyrate, Nrf2, Polystyrene microplastics, Rumen microbiota, Spermatogenesis

## Abstract

**Background:**

Microplastics are emerging environmental contaminants with male reproductive toxicity; however, their effects on ruminants and underlying mechanisms remain poorly understood.

**Results:**

In this study, male Hu lambs were exposed to dietary polystyrene microplastics (PS-MPs) at 0, 75, and 150 mg/kg diet for 120 d, followed by a 180-d recovery period. PS-MPs exposure caused persistent testicular injury, characterized by disrupted seminiferous architecture and enhanced germ cell apoptosis. Single-cell transcriptomic analysis revealed impaired spermatogonial differentiation, accompanied by suppression of Nrf2-associated antioxidant responses. Metabolomic profiling further identified dysregulation of glutathione metabolism in both testis and plasma. Importantly, PS-MPs were not detected in testicular tissue, suggesting that detectable direct particle accumulation was unlikely to be the primary driver of toxicity. Instead, rumen analyses showed that PS-MPs altered microbial composition, notably reducing butyrate-producing bacteria, accompanied by sustained decreases in ruminal butyrate and circulating β-hydroxybutyrate (BHB). To investigate the potential involvement of rumen-derived butyrate in PS-MPs–induced testicular toxicity, sodium butyrate was supplemented to PS-MPs–exposed lambs. Dynamic analyses showed that ruminal butyrate recovered first, followed by circulating BHB, then systemic inflammation and oxidative status, and finally testicular damage was alleviated with restoration of Nrf2 signaling.

**Conclusions:**

These findings suggest that PS-MPs exposure disrupts spermatogenesis in sheep through rumen-derived butyrate alterations rather than detectable direct tissue accumulation. This study provides insight into microplastic-induced testicular toxicity and supports the rumen microbiota–metabolite axis as a potential mechanistic link underlying distal organ toxicity in ruminants.

**Supplementary Information:**

The online version contains supplementary material available at https://doi.org/10.1186/s40104-026-01490-z.

## Introduction

Microplastics (MPs) are ubiquitous and persistent environmental contaminants that pose increasing threats to ecosystem stability and public health [1]. In terrestrial ecosystems, ruminants are continuously exposed to MPs via forage, feed, and drinking water [2]. MPs have been detected in sheep feces [3], ruminant gastrointestinal tracts [4], and edible tissues of cattle and sheep intended for human consumption [5]. These reports raise concerns regarding the potential impacts of microplastic pollution on ruminant health and welfare, particularly male reproductive health.

Polystyrene microplastics (PS-MPs) have been detected in bull epididymal sperm and shown to impair fertilization capacity [6]. Similarly, MPs detected in human testes and seminal plasma have been negatively associated with sperm concentration and testicular mass [7, 8]. In rodent models, prenatal and postnatal PS-MPs exposure has been shown to disrupt steroidogenesis, impair mitochondrial function, compromise blood–testis barrier integrity, and reduce male fertility [9]. Despite accumulating evidence from laboratory species and humans, whether chronic MPs exposure compromises male reproductive function in ruminants, and by which mechanisms, remains largely unknown.

Growing evidence indicates that the gut microbiota and its metabolites regulate testicular physiology through the gut–testis axis [1, 10]. Microbiota-derived metabolites are increasingly recognized as regulators of spermatogenesis and testicular homeostasis through pathways involving vitamin A metabolism, polyamine homeostasis, and inosine signaling [11–13]. In mice, MPs-induced gut dysbiosis has been shown to trigger reproductive dysfunction, which can be ameliorated by fecal microbiota transplantation [14]. However, because ruminants have distinct digestive physiology, this regulatory axis may depend more on rumen microbial metabolism than on the intestinal mechanisms described in monogastric species.

The rumen is a specialized anaerobic fermentation chamber that generates large quantities of short-chain fatty acids (SCFAs), particularly butyrate, which contribute to host energy supply, redox balance, and systemic metabolic homeostasis [15]. Prolonged feed retention and rumination behavior may facilitate the retention of MPs in the rumen and aggravate microbial ecosystem disturbances. Whether chronic MPs exposure disrupts rumen microbial metabolism and thereby contributes to impaired testicular function remains an important unresolved question.

Polystyrene (PS), a major component of agricultural plastics such as mulch films, is widely used in livestock production systems. Surveys of Chinese livestock farms have reported manure MP concentrations of 8–40 particles/L, with PS, polyethylene (PE), and polypropylene (PP) collectively accounting for more than 80% of detected polymers [16]. Because sheep are both an important food-producing species and a valuable genetic resource, MPs-induced reproductive dysfunction may pose underestimated risks to livestock productivity, breeding sustainability, and long-term genetic improvement. In the present study, we established a chronic PS-MPs exposure model in male sheep using controlled dietary exposure levels informed by ruminant feed contamination data, and we integrated multi-omics approaches to evaluate testicular toxicity. Notably, PS-MPs were not detected in testicular tissue, indicating that detectable direct microplastic accumulation was unlikely to be the primary explanation for reproductive toxicity. We therefore hypothesized that PS-MPs may impair spermatogenesis indirectly by disrupting ruminal microbial homeostasis and butyrate metabolism. By combining histology, biochemical, multi-omics, and butyrate intervention analyses, this study provides mechanistic insight into PS-MPs–induced reproductive toxicity from the perspective of a rumen microbiota–butyrate–testis axis and informs health risk assessment in ruminants.

## Materials and methods

### PS-MPs preparation

PS-MPs were purchased from Zhichuan Technology Co., Ltd. (Jiangsu, China) and identified by Fourier Transform Infrared Spectroscopy (FTIR). Particle morphology and size distribution were characterized using scanning electron microscope (EM TIC 3X, Leica, Germany) and a particle size analyzer (LS-909E, Topsizer Plus, China). Endotoxin levels in the PS-MPs preparation were measured using a chromogenic Limulus Amebocyte Lysate (LAL) assay.

### Animals and experimental design

Healthy weaned male Hu lambs aged 3 months were obtained from the Dongxiang Sheep Breeding Experimental Base of Gansu Agricultural University. The animals were housed individually in pens under controlled conditions. Experimental diets were provided twice daily at 08:00 and 18:00, with nutritional composition and feed allowance according to nutritional standards for fattening lambs [17]. Throughout the experimental period, all lambs consumed their daily feed allotment completely, with no orts remaining, and had free access to water. Before the formal experiment, all animals underwent a 10-d acclimatization period.

The study comprised two phases. The first phase involved a PS-MPs exposure-natural recovery model. A total of 36 lambs with an initial body weight of 20.64 ± 0.80 kg were randomly assigned to three groups (*n* = 12). The groups received a basal diet supplemented with 0, 75, or 150 mg/kg PS-MPs for 120 d. These exposure levels were selected based on reported microplastic contamination in ruminant feedstuffs and an approximate particle-to-mass conversion [2]. Because surveys usually report particle abundance rather than measured polymer mass, 75 mg/kg diet was defined as an environmentally informed high-end dietary exposure level, whereas 150 mg/kg diet was used as an elevated exposure level to assess dose–response effects under adverse exposure conditions. The detailed dose rationale and comparison with reported contamination levels are provided in Supplementary Methods 1.1 and Table S1. After the 120-d exposure period, six lambs from each group were randomly selected for terminal toxicity assessment. The remaining animals were switched to basal diet without added PS-MPs, initiating a 180-d natural recovery phase. These recovery groups were designated Con, NR-75, and NR-150, respectively (*n* = 6). A complete spermatogenic cycle in sheep lasts approximately 47 d [18]. The 180-d recovery period covered nearly four consecutive spermatogenic cycles. This duration was sufficient to allow multiple rounds of germ cell renewal and provided an appropriate biological window for assessing the recovery potential of spermatogenic function.

The second phase was an independent sodium butyrate (NaB) intervention trial. A total of 18 lambs with an initial body weight of 20.82 ± 0.84 kg were randomly assigned to three groups (*n* = 6): (i) the Ctrl group received a basal diet throughout the trial; (ii) the Ctrl + NaB group received a basal diet for 120 d, followed by a diet supplemented with 7 g/kg NaB for an additional 180 d; and (iii) the 150 + NaB group received a basal diet containing 150 mg/kg PS-MPs for 120 d, after which PS-MPs exposure was discontinued and the animals were fed a diet supplemented with 7 g/kg NaB for another 180 d. The NaB dose was based on previously established protocols reported to improve rumen function and gut health in sheep without inducing adverse effects [19]. Because the 150 mg/kg exposure group showed the most severe reproductive toxicity and the greatest reduction in ruminal butyrate production, this group was selected to assess the potential protective effect of NaB under the most adverse exposure conditions, rather than to compare intervention responses across different PS-MPs exposure levels. Blood and rumen fluid samples were collected every 30 d during the intervention phase to monitor dynamic changes in hormones, inflammatory markers, antioxidant indices and metabolites.

Animal numbers and sample-size reporting followed the ARRIVE 2.0 recommendations [20]. The individual lamb was considered the experimental unit. Animal numbers were determined before the experiment by considering the feasibility of a long-term large-animal study, the ethical reduction of animal use, and the need to obtain independent biological replicates for histological, biochemical, molecular, and omics analyses. Animals were randomly assigned to treatment groups using a random number generator.

### Sample collection

At the end of the experiment, all lambs were fasted for 12 h and humanely euthanized in accordance with approved institutional animal care and ethical guidelines. Jugular venous blood samples were collected immediately before euthanasia. Blood samples were allowed to clot at room temperature and then centrifuged at 4 °C and 3,000 × *g* for 10 min to obtain serum, whereas plasma samples were collected in anticoagulant-treated tubes and centrifuged under the same conditions. All serum and plasma samples were aliquoted and stored at −80 °C until analysis. After euthanasia, rumen and testicular tissues were rapidly excised and rinsed with ice-cold physiological saline. Tissue samples designated for histological examination were immediately fixed in 4% paraformaldehyde, whereas samples for electron microscopy were fixed in appropriate fixatives. Samples for molecular biology and metabolomic analyses were snap-frozen in liquid nitrogen and stored at −80 °C, whereas fresh tissues for single-cell sequencing were temporarily preserved in tissue storage solution. Rumen fluid was collected at the same time for immediate pH measurement. The remaining rumen fluid samples were snap-frozen and stored at −80 °C for subsequent volatile fatty acid (VFA) determination and metagenomic analysis.

### Determination of biochemical parameters

Follicle-stimulating hormone (FSH), luteinizing hormone (LH), testosterone (T), inhibin B (INHB), interleukin-1β (IL-1β), IL-4, IL-6, IL-10, tumor necrosis factor-α (TNF-α), total antioxidant capacity (T-AOC), superoxide dismutase (SOD), catalase (CAT), glutathione peroxidase (GSH-Px), malondialdehyde (MDA), and β-hydroxybutyrate (BHB) were quantified using commercially available assay kits according to the manufacturers’ instructions. All kits were purchased from Nanjing Jiancheng Bioengineering Institute (Nanjing, China), and detailed information is provided in Table S2.

### Morphological evaluation

Histomorphological analysis was performed on tissue samples fixed in 4% paraformaldehyde. Fixed specimens were dehydrated, embedded in paraffin, and sectioned into 5-μm slices following an established protocol [21]. Tissue sections were stained with hematoxylin and eosin (H&E) or Masson’s trichrome using commercial kits (Servicebio Technology Co., Ltd., Wuhan, China) according to the manufacturer’s instructions. At least three biological replicates were examined per experimental group. High-resolution digital whole-slide images were captured using a Leica Aperio AT2 scanner (Germany). Seminiferous tubule diameter and Johnsen score were quantified using Aperio ImageScope software (v12.3.3) [22].

Testicular tissue samples were fixed in 2.5% glutaraldehyde and post-fixed with 1% osmium tetroxide. The specimens were then dehydrated through a graded ethanol series and embedded in EPON 812 epoxy resin. Ultrathin sections (70 nm) were prepared using an ultramicrotome, collected on copper grids, and stained with uranyl acetate and lead citrate. The grids were imaged using a transmission electron microscope (TEM; Hitachi HT-7800, Japan) operating at an accelerating voltage of 80 kV.

The cauda epididymis was isolated and immersed in 1 mL of prewarmed phosphate-buffered saline (PBS; 37 °C). The tissue was finely minced with sterile scissors to allow spermatozoa to disperse into the PBS and then incubated at 37 °C for 30 min to obtain a cauda epididymal sperm suspension. Sperm concentration was determined using a hemocytometer. After thorough mixing, the sperm suspension was diluted 1:5 with PBS, and a 10-μL aliquot was loaded into the counting chamber. Sperm concentration was calculated under a light microscope using standard hemocytometer formulas and expressed as million/mL. For morphological assessment, sperm smears were prepared following the method described by Zhang et al. [12] and stained with Wright–Giemsa staining. All staining procedures were performed according to the manufacturer’s instructions (Merck KGaA, G5637-5G).

### Immunofluorescence

Prepared tissue sections were deparaffinized, rehydrated, and subjected to heat-induced antigen retrieval in sodium citrate buffer (pH 6.0). Non-specific binding sites were blocked with 5% BSA, and the sections were then incubated overnight with primary antibodies at 4 °C. The sections were then incubated with fluorescence-labeled secondary antibodies for 1 h. Information on the primary and secondary antibodies used in this study is provided in Table S3. After nuclear staining with DAPI, the specimens were coverslipped with antifade mounting medium. Images were acquired using a Zeiss LSM 880 laser scanning confocal microscope.

### Immunohistochemistry

Tissue sections were deparaffinized, rehydrated, and subjected to antigen retrieval in heated sodium citrate buffer. Sections were then immunostained according to the kit manufacturer’s instructions (Proteintech, PK10006), and the chromogenic reaction was stopped by rinsing with distilled water. After counterstaining with hematoxylin, the sections were dehydrated, cleared, and mounted with neutral balsam. Antibody information is listed in Table S3. Digital whole-slide images were acquired using a slide scanner (Leica Aperio AT2, Germany).

### Terminal deoxynucleotidyl transferase-mediated dUTP nick-end labeling (TUNEL) staining

After deparaffinization and rehydration, testicular tissue sections were permeabilized with proteinase K according to the manufacturer’s instructions. TUNEL staining was performed using a commercial TUNEL apoptosis detection kit (Abcam, ab206386). Briefly, sections were incubated with the TUNEL reaction mixture at 37 °C in a humidified chamber in the dark. After washing, nuclei were counterstained with DAPI (4′,6-diamidino-2-phenylindole). The apoptotic index was calculated as the percentage of TUNEL-positive germ cells relative to the total number of germ cells per tubule.

### Western blotting

Total proteins were extracted from testicular tissues using RIPA lysis buffer (P0013B, Beyotime), and protein concentrations were quantified using a BCA assay kit (P0012, Beyotime). Western blotting was performed according to standard procedures, as previously described [23]. After protein transfer, membranes were incubated with primary antibodies against the target protein (rabbit) and loading control (mouse), followed by incubation with species-specific HRP-conjugated secondary antibodies. Immunoreactive signals were visualized using an enhanced chemiluminescence detection system. Exposure times were adjusted to ensure that signals remained within the linear detection range. β-Actin was used as the loading control to normalize target protein expression. Band intensities were quantified using ImageJ software. Antibody information is provided in Table S3, and uncropped Western blot images are shown in Additional file 2.

### Untargeted metabolomics

Testicular and plasma samples were subjected to untargeted metabolomic profiling. Differential metabolites were screened using MetaboAnalyst 6.0 (https://www.metaboanalyst.ca) [24]. Detailed procedures are provided in Supplementary Methods 1.3.

### Single-cell RNA-sequencing and analysis

Testicular tissues from three animals were randomly selected from both the control group and the high-dose group (*n* = 3), with no sample pooling performed. Detailed procedures are provided in Supplementary Methods 1.4.

### Py-GC/MS

Detailed procedures for pyrolysis–gas chromatography/mass spectrometry (Py-GC/MS) detection and analysis are provided in Supplementary Methods 1.5. Testicular tissue samples were pretreated according to a previously described protocol [25]. Standard curves for each microplastic reference material are provided in Table S4. The content of microplastics was calculated by normalizing the detected microplastic levels to the weight of testicular samples, following the approach described by Liu et al. [26].

### Metagenomics

Detailed procedures for rumen fluid metagenomic sequencing and analysis are provided in the Supplementary Methods 1.6.

### Targeted metabolomics

The concentrations of ruminal VFAs were determined following previously described procedures [27]. Quantification was performed using an Agilent 7890B gas chromatograph equipped with a flame ionization detector (FID) and a DB-FFAP capillary column (30 m × 0.25 mm ×0.25 μm). 2-Ethylbutyric acid was used as the internal standard.

### RNA isolation and real-time quantitative PCR

Total RNA was extracted from testicular and rumen tissues using the Total RNA Kit. cDNA was synthesized using the PrimeScript RT Master Mix. Quantitative PCR (qPCR) was performed on the QuantStudio 6 Flex System using SYBR Green Mix, as previously described [28]. All kits used in this section were obtained from Thermo Fisher Scientific. Relative expression levels of target genes were calculated using the 2^−ΔΔCt^ method, with β-actin as the internal control gene. Primer sequences are listed in Table S5.

### Statistical analysis

Data are presented as the mean ± SEM. Depending on data normality, group differences were assessed using one-way ANOVA followed by Tukey’s post hoc test or the Kruskal–Wallis test followed by Dunn’s post hoc test. Testicular metabolomic data were analyzed using orthogonal partial least-squares discriminant analysis (OPLS-DA), whereas plasma metabolomic data were analyzed using principal component analysis (PCA). Metagenomic β-diversity was assessed based on binary Jaccard distances, and between-group differences were evaluated using analysis of similarities (ANOSIM). All statistical analyses were performed using GraphPad Prism version 9.0. Statistical significance was considered at *P* < 0.05.

## Results

### Characterization of PS-MPs

FTIR spectra showed the characteristic polystyrene absorption bands at 3026–3060, 1601–1451, 756 and 699 cm^−^^1^, corresponding to aromatic C–H stretching, aromatic ring C = C skeletal vibrations, and out-of-plane C–H bending of monosubstituted benzene rings, respectively, thereby confirming the identity of the particles as PS-MPs (Fig. S1A). SEM showed irregular particles with rough surfaces (Fig. S1B). Particle size showed a dominant size range of 40 to 50 μm (Fig. S1C). Endotoxin levels in the PS-MPs preparation were below the detection limit of the chromogenic LAL assay (< 0.05 EU/mL). No detectable lipopolysaccharide (LPS) contamination was detected in the PS-MPs preparation (Fig. S1D).

### PS-MPs exposure induced endocrine imbalance, oxidative–inflammatory stress, and structural damage in the testis

**Fig. 1:**
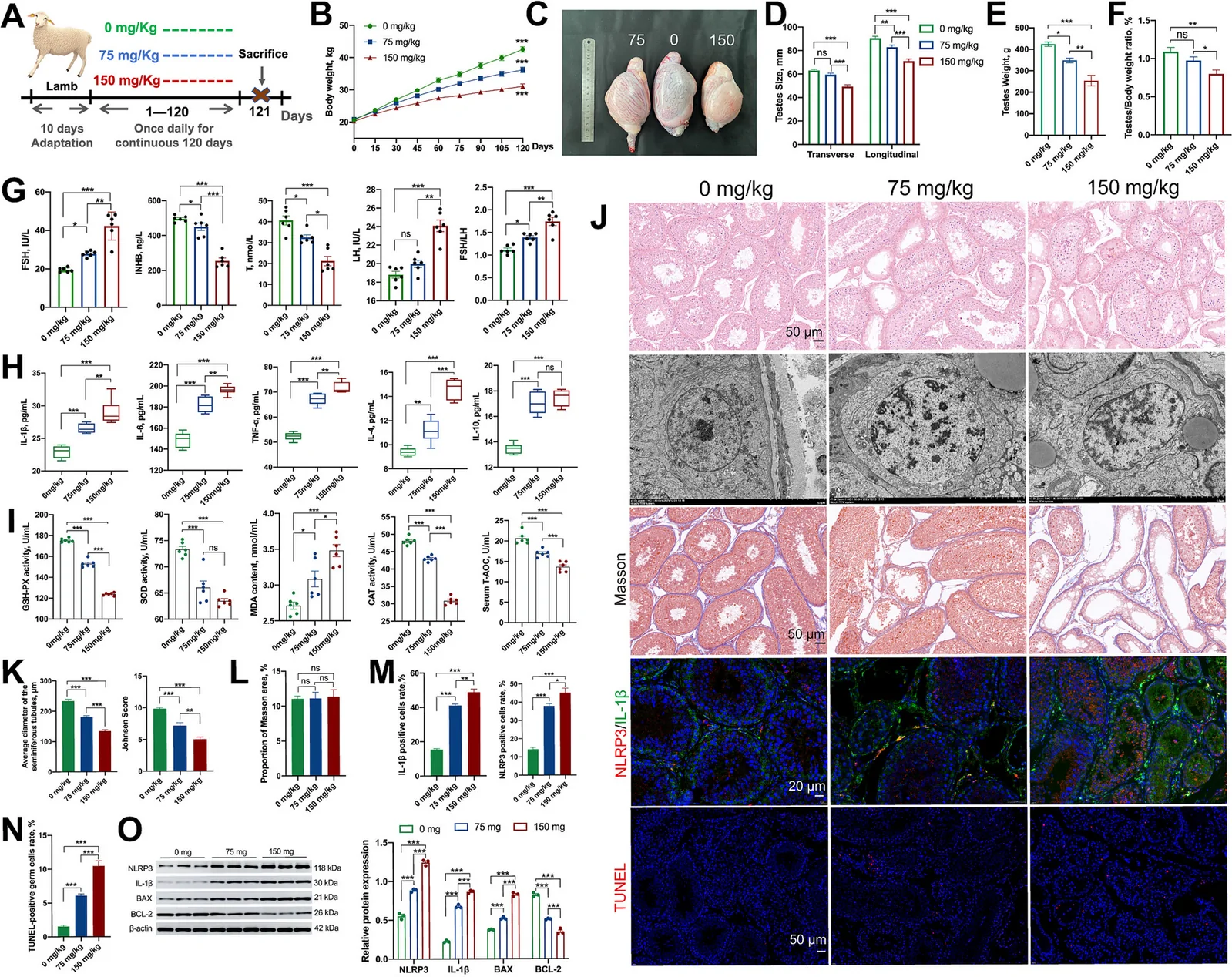
PS-MPs exposure impaired testicular structure and function and induced immune-inflammatory dysregulation. **A** Schematic diagram of the PS-MPs exposure protocol. **B** Body weight changes. **C** Gross morphology of testes. **D** Transverse and longitudinal diameters of testes. **E** Testicular weight. **F** Testis-to-body weight ratio (testis index). **G** Serum reproductive hormone levels, including FSH, INHB, T, LH, and FSH/LH ratio. **H** Serum inflammatory cytokine levels, including IL-1β, IL-6, TNF-α, IL-4, and IL-10. **I** Antioxidant enzyme activities (GSH-Px, SOD, CAT, T-AOC) and MDA content. **J** H&E staining (scale bar = 50 μm); TEM images (scale bar = 5 μm); Masson’s trichrome staining (scale bar = 50 μm); Immunofluorescence staining of NLRP3 (red) and IL-1β (green) in testicular tissue with nuclei counterstained with DAPI (blue) (scale bar = 20 μm); TUNEL staining (scale bar = 50 μm). **K** Average diameter of seminiferous tubules and Johnsen score. **L** Percentage of Masson-positive area. **M** Percentage of IL-1β- and NLRP3-positive cells. **N** Percentage of TUNEL-positive germ cells. **O** Representative Western blot images and quantitative analysis of NLRP3, IL-1β, BAX, and BCL-2 protein expression in testicular tissue. Data are presented as mean ± SEM (*n* = 6). ^ns^Not significant, ^*^P < 0.05, ^**^P < 0.01, ^***^P < 0.001

Lambs were exposed to 0, 75, or 150 mg/kg PS-MPs in the diet for 120 d (Fig. 1A). Although body weight increased over time in all groups, final body weight was lower in exposed lambs than in the 0 mg/kg group (Fig. 1B). Gross examination showed reduced testicular size in PS-MPs–exposed lambs (Fig. 1C), supported by dose-dependent decreases in transverse and longitudinal testicular diameters (Fig. 1D). Testicular weight and testis index were also decreased in the 150 mg/kg group (Fig. 1E and F).

Serum endocrine profiles were altered after PS-MPs exposure. Compared with the 0 mg/kg group, exposed lambs showed increased serum FSH, decreased INHB and T, and an increased FSH/LH ratio, whereas LH increased only in the 150 mg/kg group (Fig. 1G). Pro-inflammatory cytokines, including IL-1β, IL-6, and TNF-α, were elevated, whereas anti-inflammatory cytokines IL-4 and IL-10 were also increased (Fig. 1H). Redox homeostasis was also altered, as indicated by reduced GSH-Px and CAT activities, lower T-AOC, increased MDA, and decreased SOD activity in the 150 mg/kg group (Fig. 1I).

H&E staining showed abnormal seminiferous tubule architecture in exposed groups, including partial thinning of the seminiferous epithelium at 75 mg/kg and tubular dilation with eosinophilic degenerating germ cells at 150 mg/kg (Fig. 1J). These changes were accompanied by a reduced seminiferous tubule diameter and lower Johnsen scores (Fig. 1K). TEM showed abnormal chromatin distribution in spermatogonia from exposed animals (Fig. 1J). Masson’s trichrome staining showed no detectable increase in fibrosis (Fig. 1J and L).

Immunofluorescence showed that IL-1β was mainly localized to the basal compartment and peritubular regions, whereas NLRP3 was predominantly distributed within the seminiferous epithelium (Fig. 1J). The proportions of IL-1β- and NLRP3-positive cells increased after PS-MPs exposure (Fig. 1M). TUNEL staining showed increased apoptotic germ cells in exposed groups (Fig. 1J and N). Western blot analysis showed up-regulation of NLRP3, IL-1β, and Bax, together with down-regulation of BCL-2 (Fig. 1O).

### PS-MPs disrupted glutathione-related metabolism in the testis

**Fig. 2:**
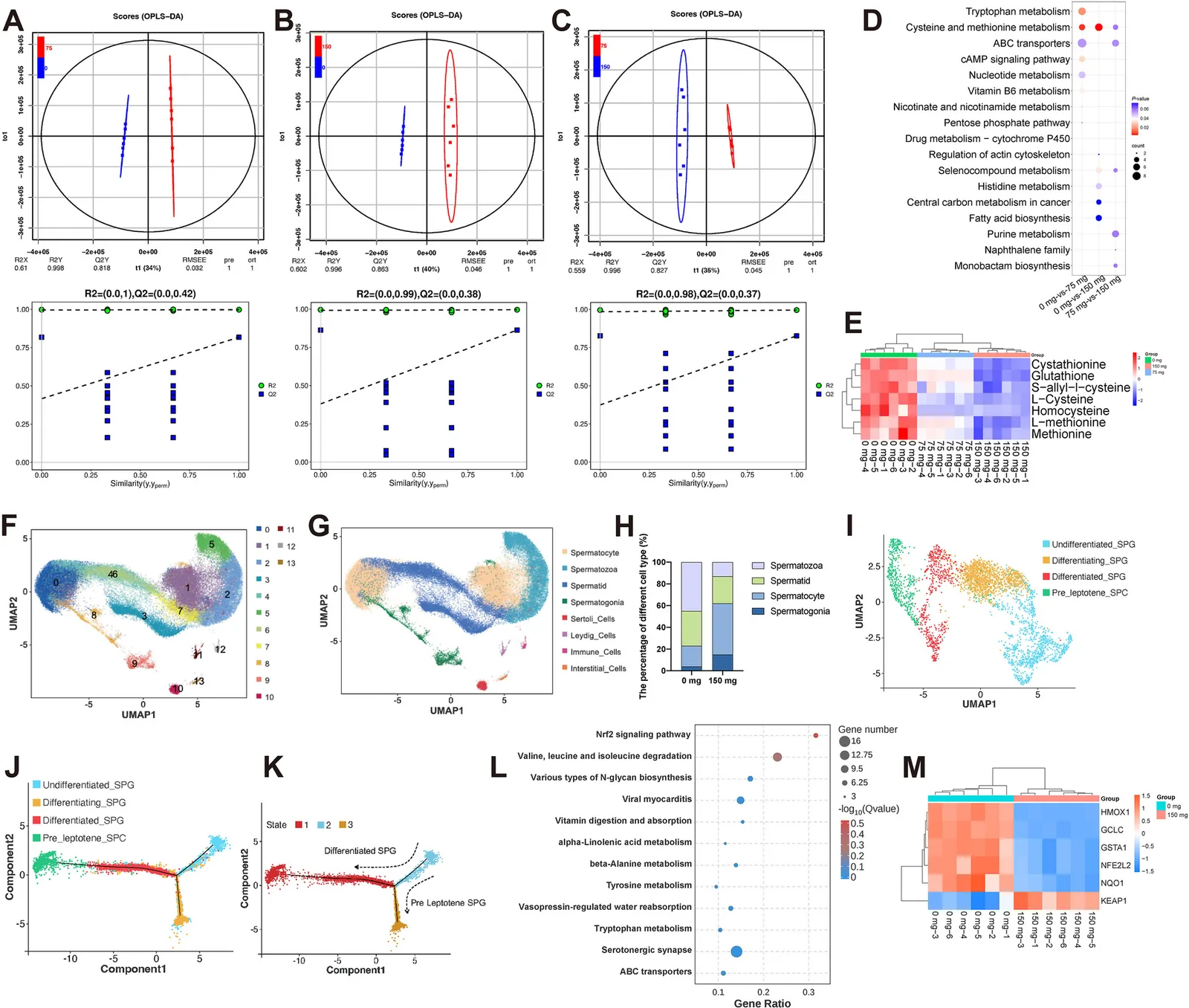
Untargeted metabolomic analysis and single-cell transcriptomic profiling of testicular tissue after PS-MPs exposure. **A–C** OPLS-DA score plots, 0 mg vs. 75 mg (**A**); 0 mg vs. 150 mg (**B**); 75 mg vs. 150 mg (**C**). Model validity was assessed by permutation testing, with R^2^Y and Q^2^ values presented. **D** KEGG pathway enrichment analysis of differential metabolites. The size of the point indicates the number of metabolites, and the color indicates the *P*-value. **E** Heatmap of metabolites involved in cysteine and methionine metabolism. Data were log2-transformed and autoscaled prior to visualization. **F **UMAP visualization showing unsupervised clustering of testicular cells. **G **Cell-type annotation based on canonical marker genes, identifying spermatogonia (SPG), spermatocytes, spermatids, Sertoli cells, Leydig cells, immune cells, and interstitial cells.** H **Proportional distribution of spermatogenic cells in the 0 mg and 150 mg groups.** I **Sub-clustering of spermatogonia revealed distinct developmental states, including undifferentiated SPG, differentiating SPG, differentiated SPG, and pre-leptotene spermatocytes. **J** and **K** Pseudotime trajectory analysis of spermatogonia differentiation.** L **KEGG pathway enrichment analysis of differentially expressed genes in spermatogonia. **M **Heatmap of differentially expressed genes involved in the Nrf2 signaling pathway

Untargeted metabolomics was performed on testicular tissue to characterize metabolic alterations. OPLS-DA showed separation in the 0 vs. 75 mg/kg, 0 vs. 150 mg/kg, and 75 vs. 150 mg/kg comparisons (R^2^Y = 0.998, Q^2^Y = 0.818; R^2^Y = 0.996, Q^2^Y = 0.863; and R^2^Y = 0.996, Q^2^Y = 0.827, respectively), and permutation testing supported the robustness of these models (Fig. 2A–C). KEGG enrichment analysis identified cysteine and methionine metabolism as one of the most affected pathways (Fig. 2D). Glutathione and methionine decreased with increasing PS-MPs dose (Fig. 2E).

### PS-MPs impaired spermatogonial differentiation and suppressed the Nrf2 signaling pathway

Single-cell RNA sequencing was performed on testicular tissue (Fig. S2A). UMAP analysis identified eight major cell populations, including spermatogonia (SPG), spermatocytes, spermatozoa, spermatids, Sertoli cells, Leydig cells, immune cells, and interstitial cells (Fig. 2F and G). Canonical marker genes supported cell annotation (Fig. S2B). Comparative analysis showed a reduced proportion of differentiated germ cells and relative accumulation of early-stage spermatogenic cells after PS-MPs exposure (Fig. 2H).

Re-clustering of spermatogonia identified four developmental states: undifferentiated SPG, differentiating SPG, differentiated SPG, and pre-leptotene spermatocytes (Pre-leptotene SPC) (Fig. 2I). Stage-specific marker expression supported annotation (Fig. S2C–F). Pseudotime analysis showed a developmental continuum from undifferentiated to differentiated states (Fig. 2J). PS-MPs exposure redistributed cells along this trajectory, with fewer cells progressing toward differentiated spermatogonia and pre-leptotene spermatocytes (Fig. 2K). Pathway analysis of differentially expressed genes revealed changes in the Nrf2 signaling pathway (Fig. 2L). Heatmap visualization showed down-regulation of *NFE2L2*, *HMOX1*, *GCLC*, *GSTA1*, and *NQO1*, together with up-regulation of *KEAP1* (Fig. 2M).

### No detectable PS-MPs accumulation in testicular tissue

**Fig. 3:**
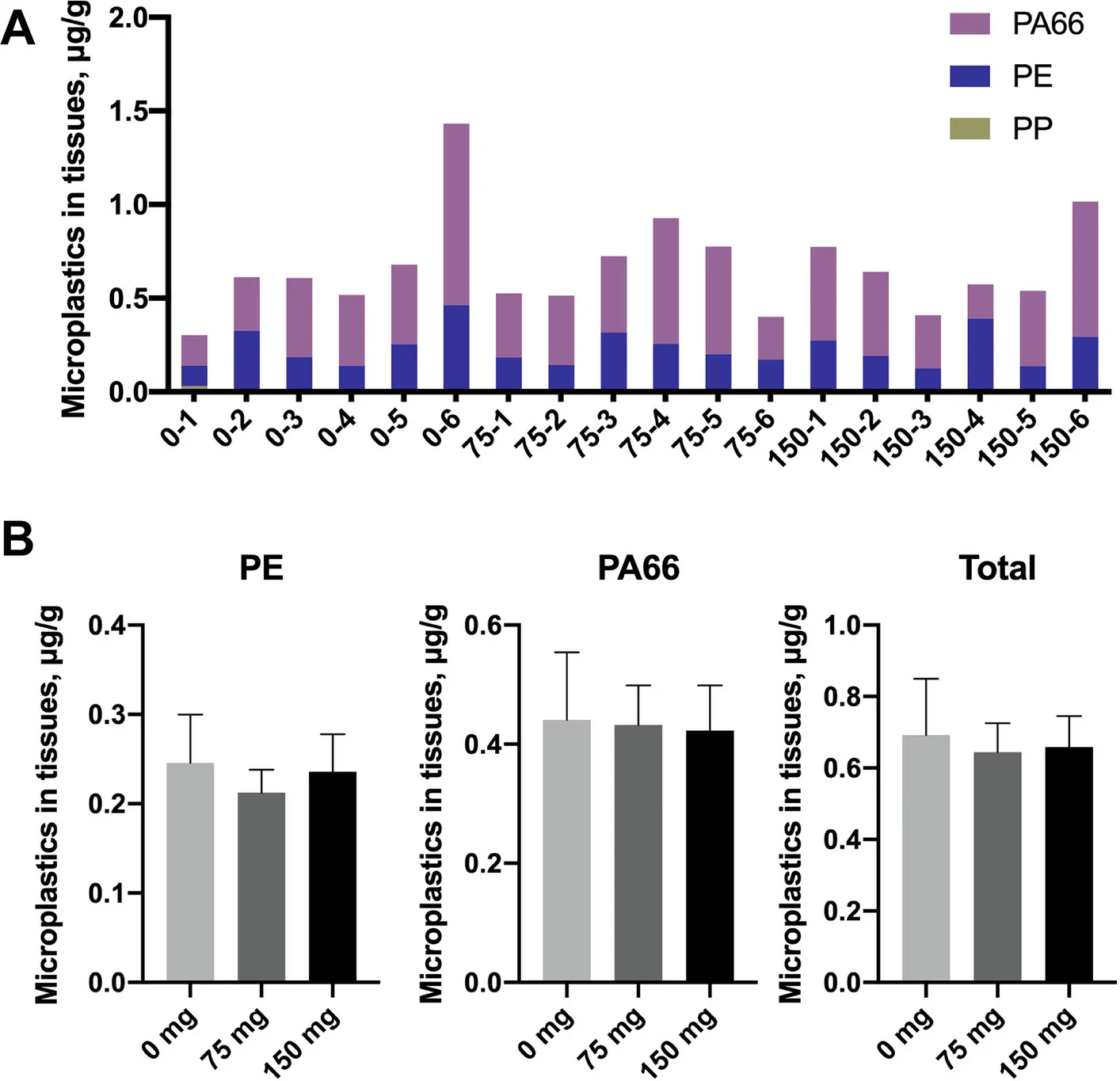
No detectable accumulation of PS-MPs in testicular tissue. **A** Microplastics content in each testicular sample. **B** Microplastics content among groups. Data are presented as mean ± SEM (*n* = 6). Differences without asterisks are not statistically significant

To examine whether PS accumulated in testicular tissue, PS content was quantified using Py-GC/MS. Within the detection limits of the method, PS was not detected in any testicular sample (Fig. 3A; Table S6), and representative chromatograms are shown in Fig. S3. PE, PA66, and total microplastics concentrations in testicular tissue did not differ among groups (Fig. 3B).

### PS-MPs disrupted systemic redox-related metabolism

**Fig. 4:**
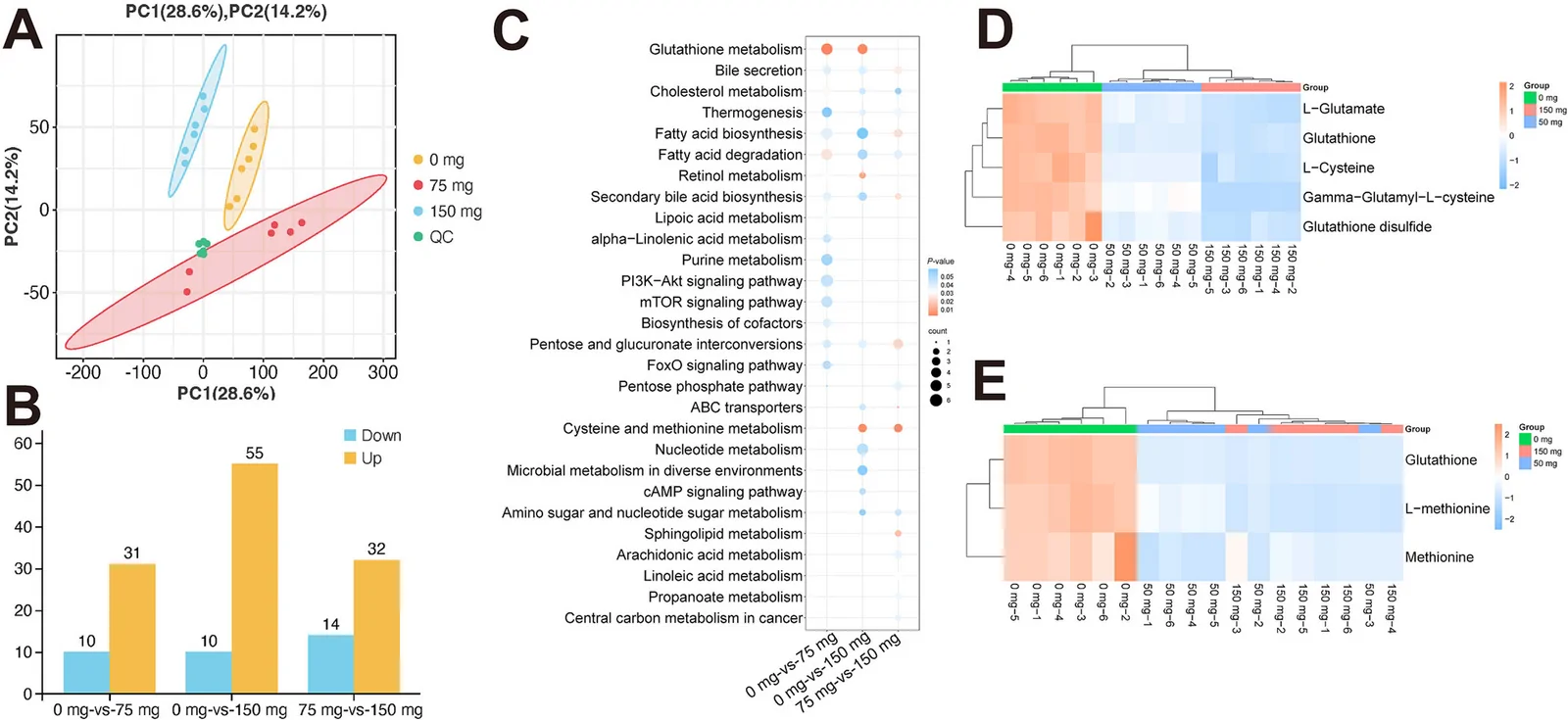
Untargeted metabolomic analysis of plasma samples following PS-MPs exposure. **A** Principal component analysis (PCA) score plot. QC indicates quality control samples. **B** Number of differential metabolites identified in group comparisons. **C** KEGG pathway enrichment analysis of differential plasma metabolites. **D** Heatmap of differential metabolites involved in glutathione metabolism. **E** Heatmap of differential metabolites involved in cysteine and methionine metabolism

Untargeted metabolomics was performed on plasma to assess systemic metabolic alterations. PCA revealed separation among the three groups, whereas QC samples clustered tightly, indicating analytical stability (Fig. 4A). More differential metabolites were detected in the 0 vs. 150 mg/kg comparison than in the 0 vs. 75 mg/kg comparison (Fig. 4B). KEGG enrichment analysis highlighted glutathione metabolism and cysteine and methionine metabolism (Fig. 4C). Key metabolites in these pathways are shown in Fig. 4D and E.

### PS-MPs disrupted ruminal microbiota, reduced butyrate production, and impaired the ruminal epithelium

**Fig. 5:**
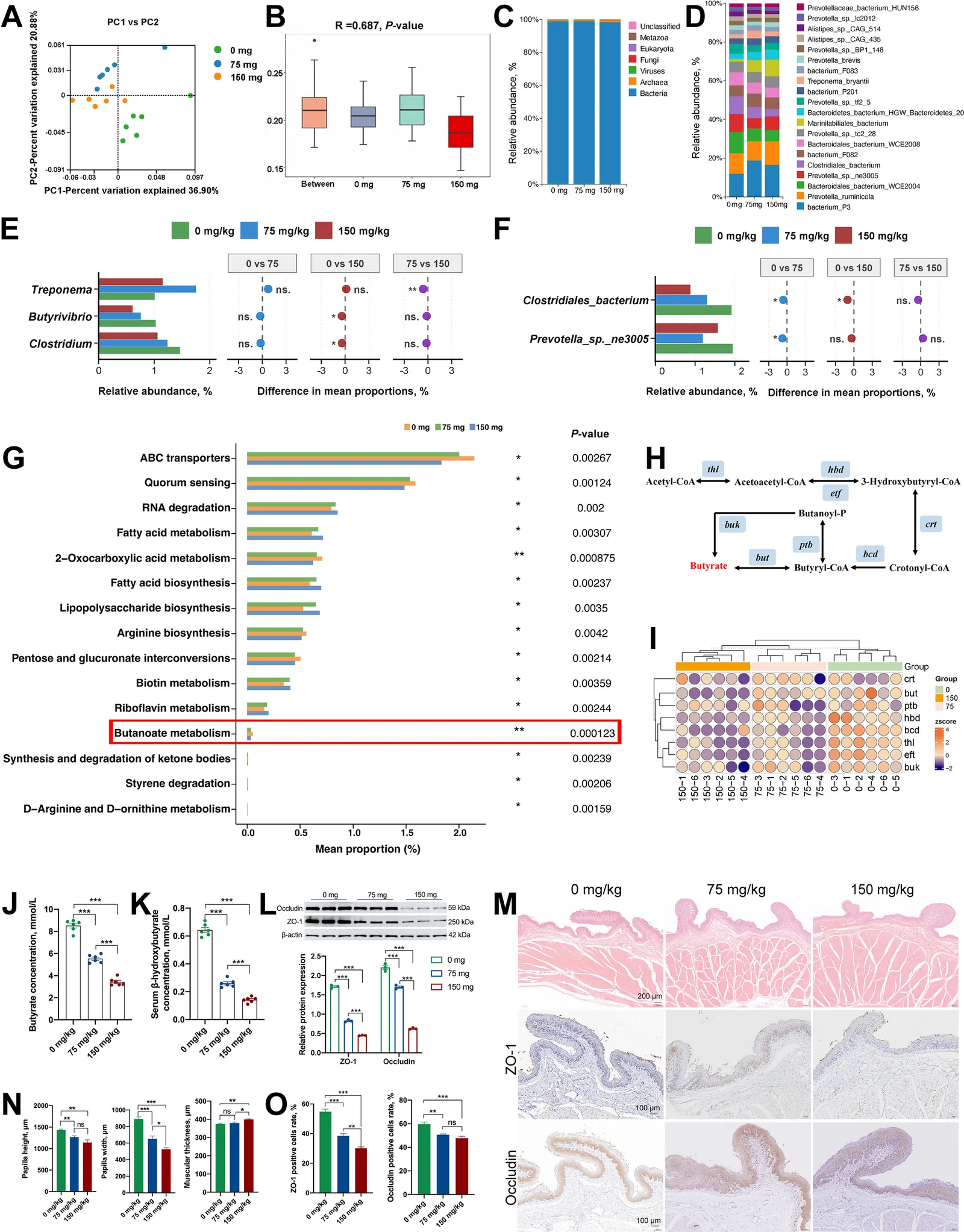
PS-MPs exposure disrupted ruminal microbiota, reduced butyrate production, and impaired epithelial tissue. **A **Principal coordinate analysis (PCoA) of the rumen microbiota. **B **Analysis of similarities showing significant differences in microbial community structure among groups (*R* = 0.687, *P* = 0.006). **C **Relative abundances of major microbial domains in rumen fluid samples. **D** Relative abundances of dominant microbial species across groups. **E** and **F** Microorganisms showing significant differences at the genus (**E**) and species (**F**) levels. **G **Differences in microbial KEGG functions among groups. **H** Butanoate metabolism. **I** Heatmap showing gene abundance in butanoate metabolism pathway. **J** Butyrate concentration in rumen fluid. **K** Serum BHB concentration. **L** Representative Western blot images and quantification of ZO-1 and occludin protein expression in rumen epithelium. **M** H&E staining of rumen epithelium (scale bar = 200 μm). Immunohistochemical staining of ZO-1 and occludin in rumen epithelium (scale bar = 100 μm). **N** Quantification of rumen papilla height, papilla width, and muscular thickness. **O** Percentage of ZO-1- and occludin-positive cells. Data are presented as mean ± SEM (*n* = 6). ^ns^Not significant, ^*^P < 0.05, ^**^P < 0.01, ^***^P < 0.001

Given the absence of detectable PS accumulation in the testis, we next investigated whether chronic PS-MPs exposure acted through the rumen. Shotgun metagenomic sequencing generated high-quality rumen microbial profiles for taxonomic and functional analyses (Table S7). β-Diversity analysis showed separation among groups in PCoA space (Fig. 5A). ANOSIM analysis showed a significant separation in microbial functional composition among groups (ANOSIM *R* = 0.687, *P* = 0.006; Fig. 5B). α-Diversity indices, including observed species, ACE, Chao1, Shannon, Simpson, and Pielou evenness, were reduced in exposed groups (Fig. S4A). Bacteria dominated the rumen microbiota (Fig. 5C). At the phylum level, the rumen microbiota remained dominated by Bacteroidota and Firmicutes (Fig. S4B), whereas the Firmicutes/Bacteroidota ratio decreased with increasing PS-MPs dose (Fig. S4C). At the genus level, *Treponema*, *Butyrivibrio*, and *Clostridium* differed among groups (Fig. S4D, Fig. 5E). At the species level, *Clostridiales bacterium* and *Prevotella* sp. ne3005 were among the altered taxa (Fig. 5D and F). LEfSe analysis showed enrichment of Firmicutes-associated lineages, including Clostridia, Eubacteriales, and Lachnospiraceae, in the 0 mg/kg group; Bacteroidetes, Marinilabiliales, and *Prevotella ruminicola* in the 75 mg/kg group; and enrichment of *Treponema* in the 150 mg/kg group (Fig. S4E). KEGG functional annotation assigned most sequences to metabolic pathways, biosynthesis of secondary metabolites, and biosynthesis of antibiotics (Fig. S4F). Comparative KEGG functional analysis identified butanoate metabolism as the most altered pathway, followed by 2-oxocarboxylic acid metabolism (Fig. 5G). Within the butanoate metabolism pathway, PS-MPs exposure reduced the relative abundance of several key functional genes, including *crt, but, ptb, hbd, bcd, thl, etf*, and *buk* (Fig. 5H and I).

Ruminal volatile fatty acids were then quantified. Ruminal butyrate concentration decreased in a dose-dependent manner (Fig. 5J), accompanied by lower total VFA levels (Table S8). Consistently, serum BHB levels were also significantly reduced in the exposed groups (Fig. 5K).

PS-MPs exposure was also associated with structural impairment of the ruminal epithelium. Western blot analysis revealed down-regulation of ZO-1 and occludin, with a greater decrease in the high-dose group (Fig. 5L). H&E staining showed shortened papillae and attenuated keratinization (Fig. 5M), and morphometric analysis showed dose-dependent reductions in papilla height, papilla width, and muscular layer thickness (Fig. 5N). Immunohistochemistry showed reduced ZO-1 and occludin positivity (Fig. 5M and O).

### Testicular and ruminal injury persists after cessation of PS-MPs exposure

After 120 d of exposure, PS-MPs were withdrawn, and animals were followed for a 180-d natural recovery period (Fig. S5A). Body weight in the NR-75 and NR-150 groups remained lower than in controls throughout recovery (Fig. S5B). At the endpoint, testes from NR-150 animals remained smaller (Fig. S5C), and transverse diameter, longitudinal diameter, testicular weight, and organ index did not return to control levels (Fig. S5D–F). Endocrine alterations also persisted, including changes in FSH, LH, INHB, T, and FSH/LH ratio (Fig. S5G). Pro-inflammatory cytokines remained elevated (Fig. S5H), whereas antioxidant indices remained altered, with lower GSH-Px, CAT, SOD, and T-AOC and higher MDA (Fig. S5I).

Testicular histological alterations also persisted. H&E staining showed reduced spermatogenic epithelial layers and tubular lumen dilation in the NR groups (Fig. S6A). Seminiferous tubule diameter and Johnsen scores remained lower than in controls (Fig. S6B and C), and TEM showed continued ultrastructural abnormalities (Fig. S6D). Masson staining showed no differences among groups (Fig. S6E and F). Pro-inflammatory cytokines, anti-inflammatory cytokines and apoptotic markers remained elevated, including increased NLRP3 and IL-1β immunofluorescence signals (Fig. S6G–I), increased TUNEL-positive germ cells (Fig. S6J and K), and up-regulated NLRP3, IL-1β, and Bax with reduced BCL-2 by Western blotting (Fig. S6L and M).

Ruminal alterations also persisted after exposure cessation. Ruminal butyrate concentration remained lower in the NR-75 and NR-150 groups than in Con group (Fig. S7A; Table S9), and circulating BHB did not normalize (Fig. S7B). Western blotting showed sustained down-regulation of ZO-1 and occludin (Fig. S7C), and histological analysis showed papillary damage with reduced papilla height and width (Fig. S7D and E). Immunohistochemistry showed weak ZO-1 and occludin staining in the NR groups (Fig. S7D and F).

### Ruminal butyrate recovery preceded systemic improvement after NaB intervention

**Fig. 6:**
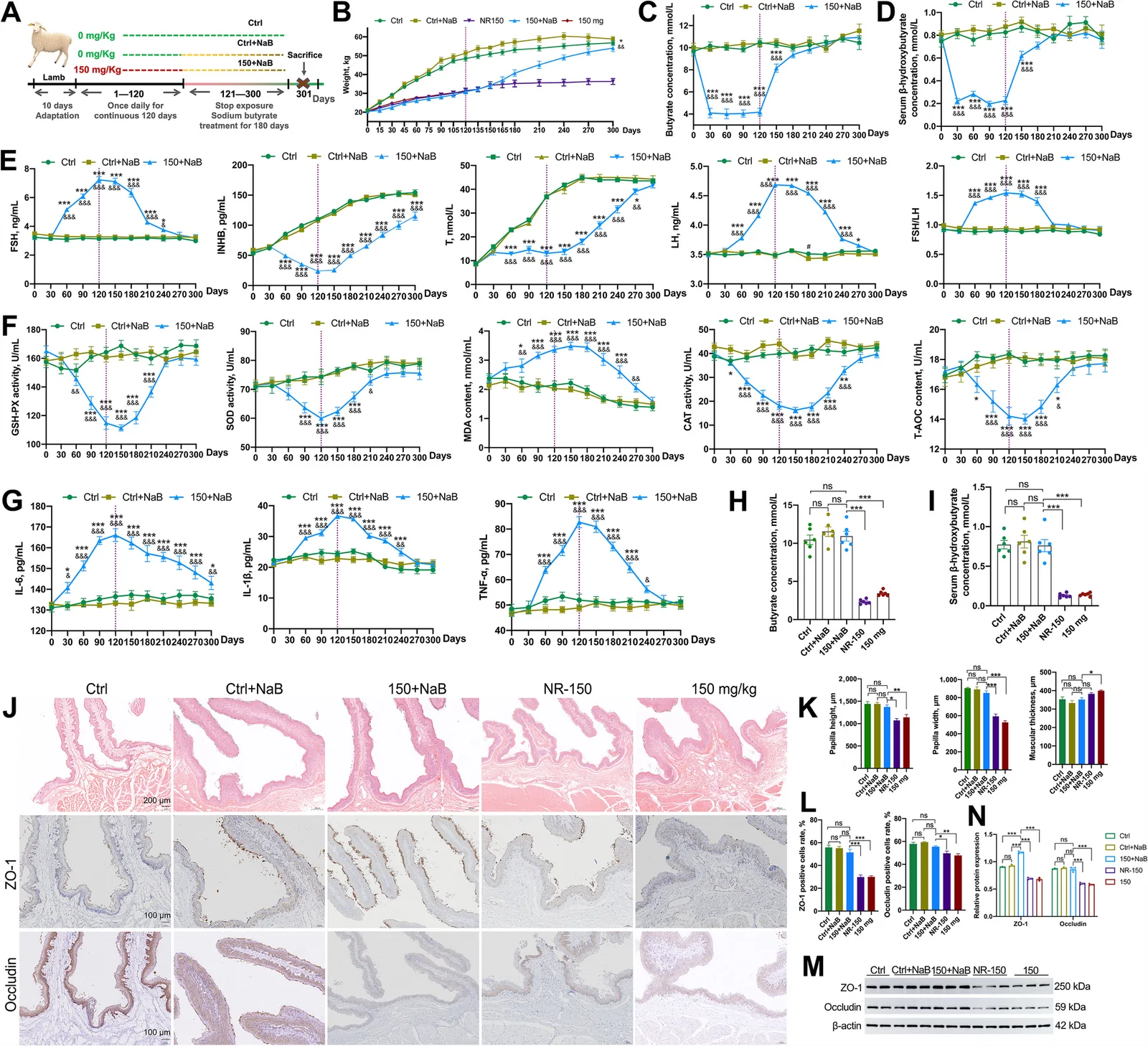
Sodium butyrate (NaB) supplementation restored ruminal function and systemic homeostasis following PS-MPs exposure.** A** Schematic illustration of the exposure-treatment experimental design. **B** Body weight changes. **C** Temporal changes in ruminal butyrate concentrations. **D** Temporal changes in serum BHB concentrations. **E** Dynamic changes in serum reproductive hormones, including FSH, INHB, testosterone (T), LH, and FSH/LH ratio. **F** Dynamic changes in serum antioxidant enzyme activities, including GSH-Px, SOD, CAT, T-AOC, and MDA content. **G** Dynamic changes in serum inflammatory cytokine concentrations, including IL-1β, IL-6, TNF-α, IL-4, and IL-10. **H** Ruminal volatile fatty acid concentrations at the endpoint. **I** Serum BHB concentration at the endpoint. **J** Representative H&E-stained ruminal epithelium sections (scale bar = 200 μm) and immunohistochemical staining of tight junction proteins ZO-1 and occludin (scale bar = 100 μm). **K** and **L** Quantitative analysis of ruminal papilla height, papilla width, and tight junction protein expression. **M** Representative Western blot images of ZO-1 and occludin protein expression. **N** Quantitative analysis of ZO-1 and occludin protein levels. Data are presented as mean ± SEM (*n* = 6). ^ns^Not significant, ^*^P < 0.05, ^**^P < 0.01, ^***^P < 0.001

To assess the involvement of ruminal butyrate deficiency, lambs exposed to 150 mg/kg PS-MPs for 120 d subsequently received dietary sodium butyrate (NaB) for 180 d (Fig. 6A). NaB supplementation increased body weight gain, although final body weight in the 150 + NaB group remained lower than in the Ctrl and Ctrl + NaB groups (Fig. 6B). Dynamic monitoring showed that ruminal butyrate increased rapidly after NaB supplementation and remained near control levels (Fig. 6C), whereas serum BHB increased by d 60 (Fig. 6D).

Endocrine parameters gradually approached control values during intervention. Serum FSH, LH, INHB, T, and the FSH/LH ratio progressively approached control values in the 150 + NaB group, with no endpoint differences from controls (Fig. 6E). In the Ctrl + NaB group, these endocrine parameters remained unchanged throughout the experiment. Antioxidant, pro-inflammatory, and anti-inflammatory indices also improved progressively: GSH-Px, SOD, CAT, and T-AOC increased, whereas MDA decreased during NaB supplementation (Fig. 6F). IL-1β and TNF-α also decreased and were no longer different from controls at the endpoint, whereas IL-6 remained elevated during supplementation (Fig. 6G). At the endpoint, ruminal butyrate and serum BHB were higher in the 150 + NaB group than in the 150 mg/kg and NR-150 groups (Fig. 6H and I; Table S10).

NaB supplementation was accompanied by restoration of ruminal epithelial structure and barrier integrity. H&E staining showed improved papillary morphology, and papilla height, papilla width, and muscular layer thickness reached levels comparable to controls (Fig. 6J and K). Immunohistochemistry showed stronger ZO-1 and occludin staining (Fig. 6J and L), and Western blot analysis showed recovery of both proteins to control levels (Fig. 6M and N).

### NaB improved local redox balance, testicular structure, and sperm parameters

At the intervention endpoint, NaB supplementation improved testicular morphology (Fig. S8A). Transverse and longitudinal testicular diameters and testicular weight were higher in the 150 + NaB group than in the 150 mg/kg and NR-150 groups and were comparable to Ctrl values (Fig. S8B and C). Testis index did not differ among groups (Fig. S8D). Testicular SOD, CAT, GSH-Px, and T-AOC activities increased, whereas MDA decreased in the 150 + NaB group (Fig. S8E).

Histological changes also improved. H&E staining showed improved seminiferous epithelium in the 150 + NaB group, accompanied by higher seminiferous tubule diameter and Johnsen scores (Fig. S8F and G). TEM showed improved nuclear morphology in spermatogonia compared with the 150 mg/kg and NR-150 groups (Fig. S8F). Masson’s trichrome staining remained unchanged among groups (Fig. S8F and H). NLRP3 and IL-1β immunofluorescence was reduced in the 150 + NaB group (Fig. S8F and I), and TUNEL staining showed fewer apoptotic germ cells (Fig. S8F and J).

Epididymal sperm output and morphology improved after NaB supplementation. H&E staining of the cauda epididymis showed densely packed spermatozoa in the Ctrl, Ctrl + NaB, and 150 + NaB groups, whereas significantly fewer sperm were observed in the 150 mg/kg and NR-150 groups (Fig. S9A). Cauda epididymal sperm concentration decreased after PS-MPs exposure and remained low after natural recovery, but increased toward control levels after NaB supplementation (Fig. S9B). Sperm smear analysis showed a higher proportion of abnormal sperm in the 150 mg/kg and NR-150 groups, including head and tail defects, tail curling, and breakage, whereas NaB reduced the abnormal sperm rate (Fig. S9C and D).

### NaB supplementation was accompanied by restoration of testicular Nrf2-related antioxidant signaling

**Fig. 7:**
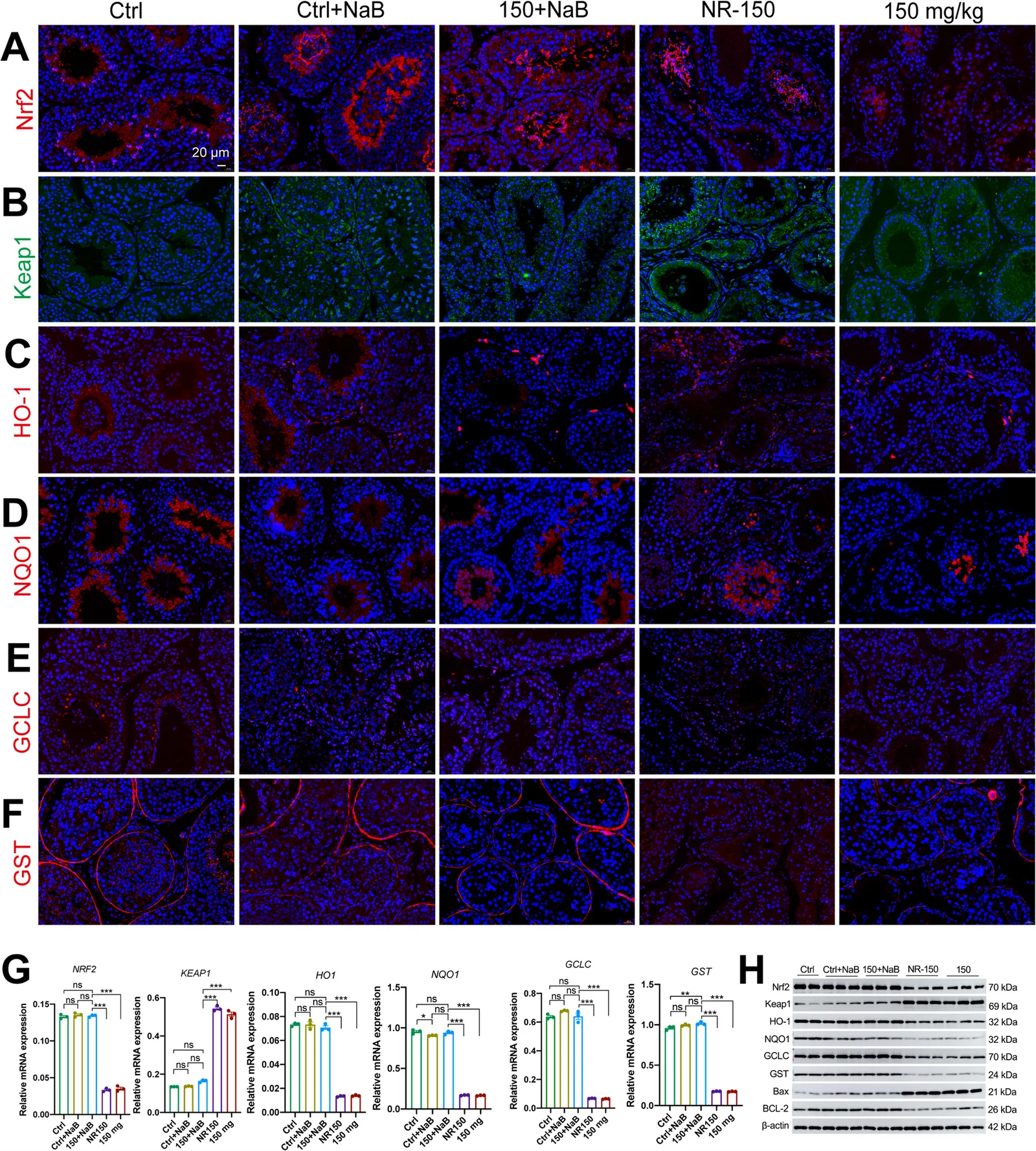
Reactivation of the Nrf2 signaling pathway in testicular tissue following sodium butyrate intervention. **A–F** Immunofluorescence staining of Nrf2 (**A**), Keap1 (**B**), HO-1 (**C**), NQO1 (**D**), GCLC (**E**), and GST (**F**) in testicular sections (scale bar = 20 μm). **G** Relative mRNA expression levels of *NRF2, KEAP1, HO-1, NQO1, GCLC*, and *GST* in testicular tissue. **H** Representative Western blot images of Nrf2, Keap1, HO-1, NQO1, GCLC, GST, BAX, and BCL-2 in testicular tissue. Data are presented as mean ± SEM. ^ns^Not significant, ^*^P < 0.05, ^**^P < 0.01, ^***^P < 0.001

Immunofluorescence showed that Nrf2, Keap1, HO-1, NQO1, GCLC, and GST were mainly localized in seminiferous epithelial regions (Fig. 7A–F). The positive expression ratios of these markers in the 150 + NaB group did not differ from those in the Ctrl group (Fig. S10A). RT-qPCR analysis further showed that *NRF2*, *KEAP1*, *HO-1*,* NQO1*,* GCLC*, and *GST* returned to control levels in the 150 + NaB group (Fig. 7G). Western blot analysis showed increased Nrf2 and downstream effectors, reduced Keap1 expression, and decreased Bax/BCL-2 ratio after NaB supplementation (Fig. 7H; Fig. S10B).

## Discussion

**Fig. 8:**
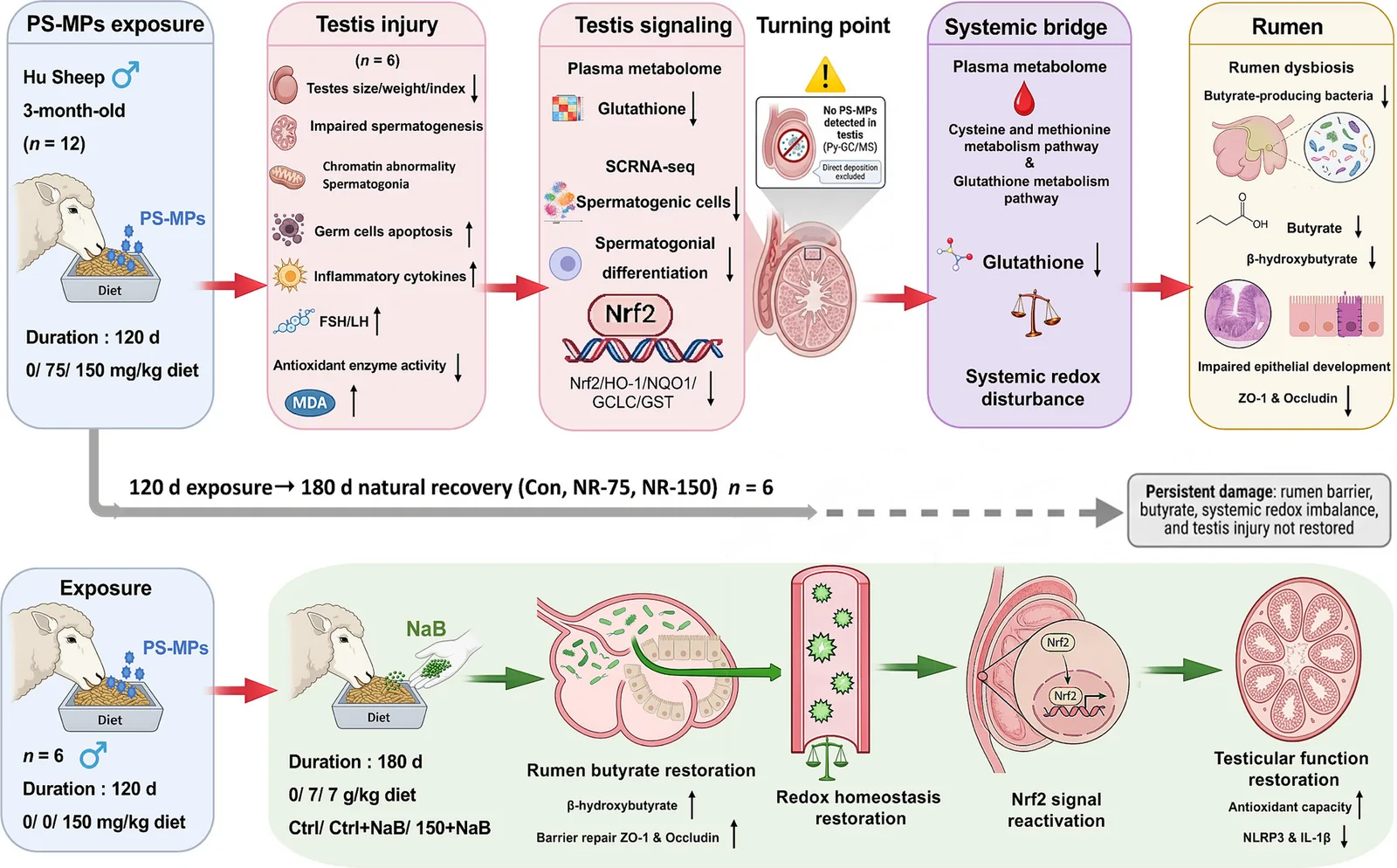
Chronic PS-MPs exposure impairs spermatogenesis by disrupting ruminal butyrate production. PS-MPs exposure-induced ruminal microbial dysbiosis is associated with insufficient ruminal butyrate and circulating BHB, which consequently induces systemic redox imbalance and suppresses Nrf2-associated antioxidant defense in the testes, ultimately resulting in testicular dysfunction. However, sodium butyrate (NaB) intervention significantly restores both ruminal and testicular function, supporting the critical role of ruminal butyrate in PS-MPs-induced toxicity

Microplastics pose potential systemic risks to both human and animal health. Although accumulating evidence links microplastic exposure to male reproductive dysfunction and declining fertility [29, 30], their risks and underlying mechanisms in ruminants remain unclear. In the present study, PS-MPs exposure impaired testicular function, accompanied by disruption of ruminal microbial homeostasis, reduced ruminal butyrate and circulating BHB, systemic redox imbalance, and suppression of Nrf2-associated antioxidant signaling. Instead, NaB intervention improved ruminal and testicular indices, supporting the involvement of ruminal butyrate in PS-MPs-associated toxicity (Fig. 8).

Consistent with previous reports, microplastic-induced male reproductive toxicity is often accompanied by oxidative stress and inflammatory responses, and impaired spermatogenesis [30, 31]. In this study, chronic PS-MPs exposure induced structural and functional testicular damage, accompanied by activation of NLRP3 and IL-1β signaling and elevated germ-cell apoptosis. Metabolomic analyses showed alterations in cysteine and methionine metabolism after PS-MPs exposure. As cysteine is the rate-limiting substrate for glutathione (GSH) synthesis, its depletion directly exhausts intracellular GSH pools and compromises the activities of key antioxidant enzymes, including GPx and SOD [32]. In the testes, GSH functions as an antioxidant cofactor and help protect germ-cell membrane lipids from peroxidation and ferroptosis [33]. Single-cell RNA sequencing further revealed that PS-MPs exposure impeded spermatogonia differentiation, leading to spermatogenic arrest, while concomitant suppression of the Nrf2 pathway. These findings are consistent with observations in rodent models, in which micro- and nanoplastics induced metabolic dysregulation, oxidative damage, and spermatogenic impairment [9, 34].

Although testicular injury was evident at the exposure endpoint, polystyrene (PS) was not detected in testicular tissue within the detection limits of Py-GC/MS. This apparent discrepancy should be interpreted in relation to previous studies reporting MPs or PS-MPs in bull epididymal sperm, human testes, human seminal fluid, and mouse models [6–9]. Several methodological and biological factors may account for these differences. First, the biological matrices differed across studies. Previous studies detected MPs mainly in epididymal sperm, seminal fluid, or human reproductive tissues, whereas the present study quantified polymer content in sheep testicular tissue [6–8]. Detection in semen or epididymal sperm does not necessarily indicate measurable accumulation in the testicular parenchyma. Second, exposure scenarios differed. Human studies reflect long-term, mixed-polymer, multi-route environmental exposure, whereas rodent studies often involve sensitive prenatal and postnatal exposure windows [7–9]. In contrast, the present study used a controlled dietary exposure model in post-weaning lambs. Third, particle size and ruminant digestive physiology may influence tissue translocation. The PS-MPs used here were predominantly 40–50 μm, whereas smaller particles, particularly submicron PS beads, may have greater potential for biological interaction and systemic translocation [6]. Consistent with interpretation, oral kinetic evidence in sheep showed that most radiolabeled PS-MPs were excreted in feces with negligible systemic redistribution [35]. Finally, analytical methods differed. Py-GC/MS provides polymer mass-based quantification, whereas Raman-based methods used in human studies identify individual particles [7, 8]. Therefore, the negative finding in the present study should be interpreted “not detected within the detection limits of Py-GC/MS” rather than as evidence for the complete absence of PS particles. Under the present exposure conditions, testicular injury was unlikely to be primarily explained by detectable direct PS accumulation.

Plasma metabolomics also revealed perturbations in glutathione metabolism, suggesting that redox imbalance was systemic rather than confined to the testis. Previous studies have shown that microplastics can induce oxidative stress, inflammation, and metabolic reprogramming, thereby promoting the activation of pathways associated with biological aging [36]. Recent evidence further demonstrates that polystyrene exposure drives selective enrichment of gut microbiota, accompanied by disruptions in redox homeostasis and hormone metabolism [37]. Collectively, these systemic alterations may represent a mechanistic bridge linking exposure to adverse reproductive outcomes.

Given the central role of the rumen in nutrient utilization, metabolic homeostasis, and mucosal immunity in ruminants, we further examined upstream ruminal alterations. PS-MPs exposure reduced the abundance of butyrate-producing bacteria, decreased ruminal butyrate concentration, impaired ruminal epithelium and downregulated barrier-related proteins. Both in vivo and in vitro studies indicate that microplastics can directly interact with rumen microbiota, disrupt ruminal fermentation, and reshape VFA profiles [38, 39]. In ruminants, more than 90% of ruminal butyrate is metabolized into BHB within the rumen epithelium and subsequently released into the portal circulation [40]. Therefore, reduced butyrate production may limit peripheral BHB availability and contribute to systemic oxidative stress and immune-inflammatory dysregulation.

Butyrate and BHB are not only energy substrates but also key signaling molecules that regulate rumen epithelial development and immune homeostasis in ruminants [41]. As endogenous histone deacetylase (HDAC) inhibitors, these metabolites promote Nrf2 nuclear translocation through GPR109A-AMPK and GSK-3β pathways and upregulate downstream antioxidant genes, including HO-1, NQO1, GCLC, and GST [42]. In parallel, BHB has been reported to suppress NLRP3 inflammasome activation and reduce IL-1β release [43]. Butyrate is also an activator of Nrf2 [43]. Previous studies have demonstrated that butyrate enhances Nrf2 expression in intestinal epithelial cells [44], hepatocytes [45], and dairy goat models of subacute ruminal acidosis [46], with concomitant activation of antioxidant programs and suppression of inflammatory responses. Nrf2 is widely expressed in the testis and is considered a therapeutic target for male infertility because of its role in antioxidant defense against heat stress, environmental toxicants, and aging [47]. Impaired Nrf2 function has been associated with testicular weight loss, reduced testosterone levels, and disrupted spermatogenic architecture [48], whereas Nrf2 activation may inhibit ferroptosis and support spermatogenesis [49]. In addition, BHB serves as an energy substrate for spermatozoa [50] and a protective factor for Leydig cells [51], further supporting its relevance to male reproduction. Thus, the persistent testicular injury after PS-MPs exposure may be attributable to insufficient ruminal butyrate, which may disrupt upstream metabolic signaling, weaken Nrf2-associated antioxidant defenses, and amplify oxidative–inflammatory responses.

Our findings suggest that NaB supplementation effectively alleviates PS-MPs–induced testicular dysfunction. After cessation of exposure, NaB supplementation was accompanied by early recovery of ruminal butyrate and circulating BHB, followed by attenuation of systemic oxidative–inflammatory imbalance, restoration of testicular Nrf2-related antioxidant defenses, and partial amelioration of testicular dysfunction. This temporal sequence supports that butyrate insufficiency may act as an upstream event in the development of testicular dysfunction. Consistently, Liu et al. reported that high-fat diet-induced reductions in butyrate were associated with decreased testosterone levels and impaired sperm quality, whereas butyrate supplementation reversed these alterations [52]. Collectively, these findings support an important role for rumen-derived butyrate metabolic signaling in PS-MPs-induced testicular injury.

Nevertheless, several limitations of this study should be acknowledged. Although the multi-omics data and NaB intervention support a role for rumen-derived butyrate signaling, this study did not include rumen microbiota transplantation or selective depletion of butyrate-producing bacteria. Thus, whether PS-MPs-altered rumen microbiota alone can initiate testicular injury remains to be determined. In addition, future studies using pharmacological inhibition such as ML385 or genetic suppression of Nrf2 will be necessary to determine whether NaB-mediated protection is abolished when Nrf2 signaling is blocked. Finally, although NaB supplementation improved cauda epididymal sperm concentration and morphology, direct fertility outcomes were not assessed. Therefore, future studies using artificial insemination or controlled mating trials are required to evaluate functional fertility.

## Conclusion

In conclusion, chronic exposure to PS-MPs impaired spermatogenesis in sheep and was associated with persistent disruption of ruminal microbial homeostasis, reduced ruminal butyrate and circulating BHB, systemic redox imbalance, and suppression of Nrf2-associated antioxidant signaling. The absence of detectable polystyrene accumulation in testicular tissue, together with the protective effects of NaB supplementation, supports a central role for rumen-derived butyrate signaling in PS-MPs–associated testicular toxicity. These findings support the rumen microbiota–metabolite axis as a potential mechanistic link between chronic microplastic exposure and male reproductive dysfunction in ruminants.

## Supplementary Information

Additional file 1: Supplementary Methods 1.1. Dose Rationale. Supplementary Methods 1.2. Endotoxin detection of PS-MPs. Supplementary Methods 1.3. Untargeted metabolomics. Supplementary Methods 1.4. Single cell RNA-Sequencing and Analyses. Supplementary Methods 1.5. Py-GC/MS. Supplementary Methods 1.6. Metagenomics. Fig. S1. Characterization of PS-MPs. Fig. S2. Marker gene expression and validation of spermatogenic subpopulations in sheep testes. Fig. S3. Py-GC/MS chromatograms. Fig. S4. The effects of PS-MPs exposure on rumen microbial diversity and function. Fig. S5. Systemic hormonal, inflammatory, and antioxidant disturbances failed to recover after cessation of exposure. Fig. S6. PS-MPs–induced testicular histopathological damage, inflammation, and apoptosis failed to recover naturally. Fig. S7. Ruminal butyrate depletion and epithelial tissue damage persisted after natural recovery. Fig. S8. NaB supplementation significantly restored testicular morphology and suppressed inflammation and apoptosis following PS-MPs exposure. Fig. S9. Histological and seminal examinations of cauda epididymis. Fig. S10. Quantification of Nrf2 pathway and apoptosis-related proteins in testicular tissue. Table S1. Mapping of reported ruminant feed microplastic contamination to approximate mass-based estimates and experimental PS-MPs doses. Table S2. Kit information. Table S3. Primary and secondary antibody information. Table S4. Py-GC/MS calibration curves of polymers. Table S5. Primer sequences. Table S6. Microplastics profiles in testicular samples. Table S7. Sample sequencing data quality control and assembly results. Table S8. Ruminal volatile fatty acid profiles and pH in sheep exposed to PS-MPs. Table S9. Natural recovery of ruminal volatile fatty acids and pH after cessation of PS-MPs exposure in sheep. Table S10. Changes in ruminal volatile fatty acids and pH in sheep supplemented with sodium butyrate after cessation of exposure

Additional file 2: Uncropped Western blot images.

## Data Availability

The datasets used during the current study are available from the corresponding author on reasonable request.

## Abbreviations

BHB: β-Hydroxybutyrate
CAT: Catalase
FSH: Follicle-stimulating hormone
FTIR: Fourier transform infrared spectroscopy
GSH-Px: Glutathione peroxidase
IL-1β: Interleukin-1 beta
IL-4: Interleukin-4
IL-6: Interleukin-6
IL-10: Interleukin-10
INHB: Inhibin B
LH: Luteinizing hormone
MDA: Malondialdehyde
MPs: Microplastics
PS-MPs: Polystyrene microplastics
SCFAs: Short-chain fatty acids
SOD: Superoxide dismutase
T: Testosterone
T-AOC: Total antioxidant capacity
TNF-α: Tumor necrosis factor-alpha
VFA: Volatile fatty acid
