# Supplementary material for: Polystyrene microplastics impair sheep spermatogenesis by disrupting rumen microbiota-derived butyrate signaling
Source: J Anim Sci Biotechnol. 2026 Sep 9;17:177. doi: 10.1186/s40104-026-01490-z (PMC13555869; doi:10.1186/s40104-026-01490-z)
Supplement: Supplementary file 1 [file 40104_2026_1490_MOESM2_ESM.pdf]

# Fig. 1

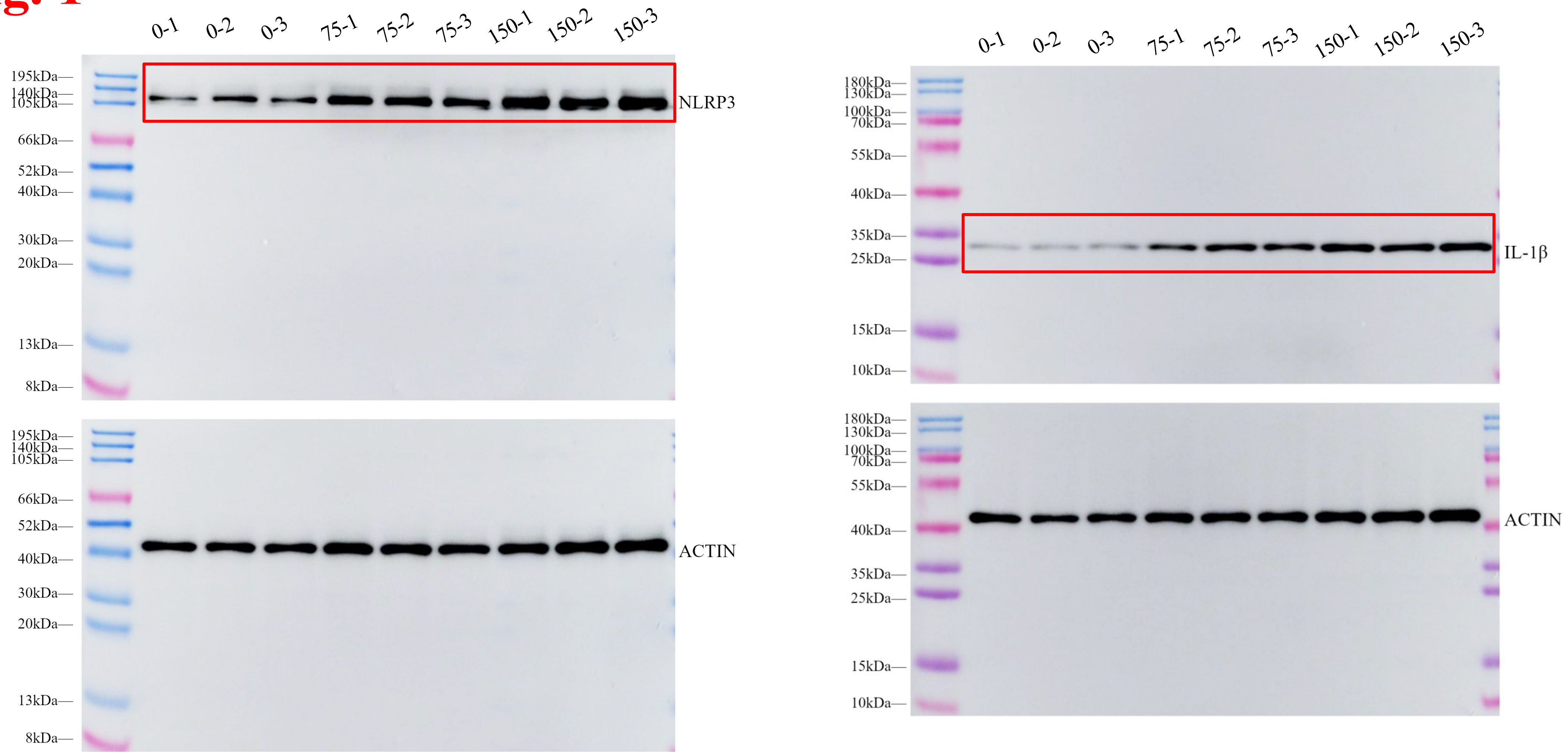
0-1
0-2
0-3
75-1
75-2
75-3
150-1
150-2
150-3
195kDa
140kDa
105kDa
66kDa
52kDa
40kDa
30kDa
20kDa
13kDa
8kDa
NLRP3
ACTIN
180kDa
130kDa
100kDa
70kDa
55kDa
35kDa
25kDa
15kDa
10kDa
IL-1β

**Fig. 1**

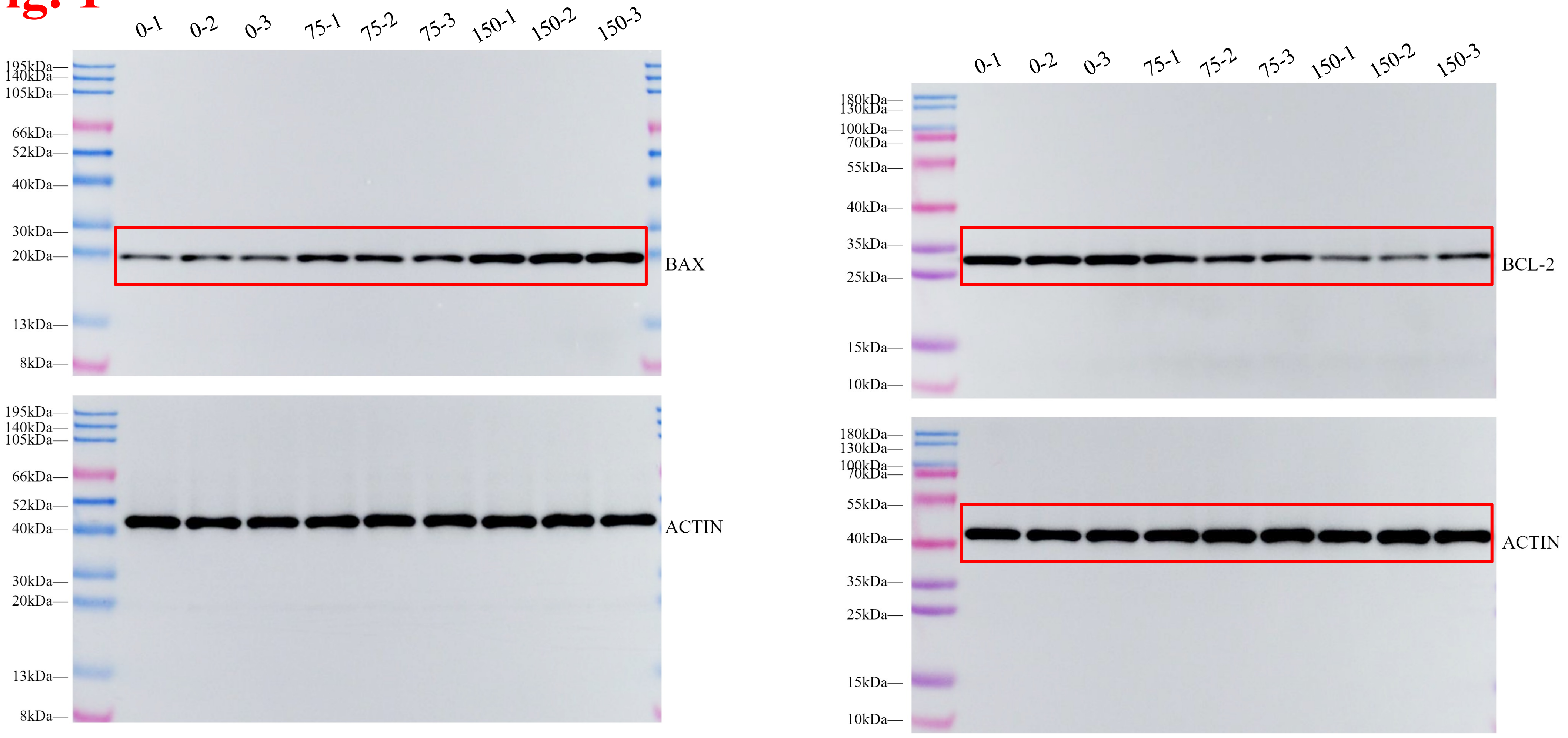

0-1
0-2
0-3
75-1
75-2
75-3
150-1
150-2
150-3
195kDa
140kDa
105kDa
66kDa
52kDa
40kDa
30kDa
20kDa
13kDa
8kDa
BAX
ACTIN
180kDa
130kDa
100kDa
70kDa
55kDa
40kDa
35kDa
25kDa
15kDa
10kDa
BCL-2
ACTIN

# Fig. 5

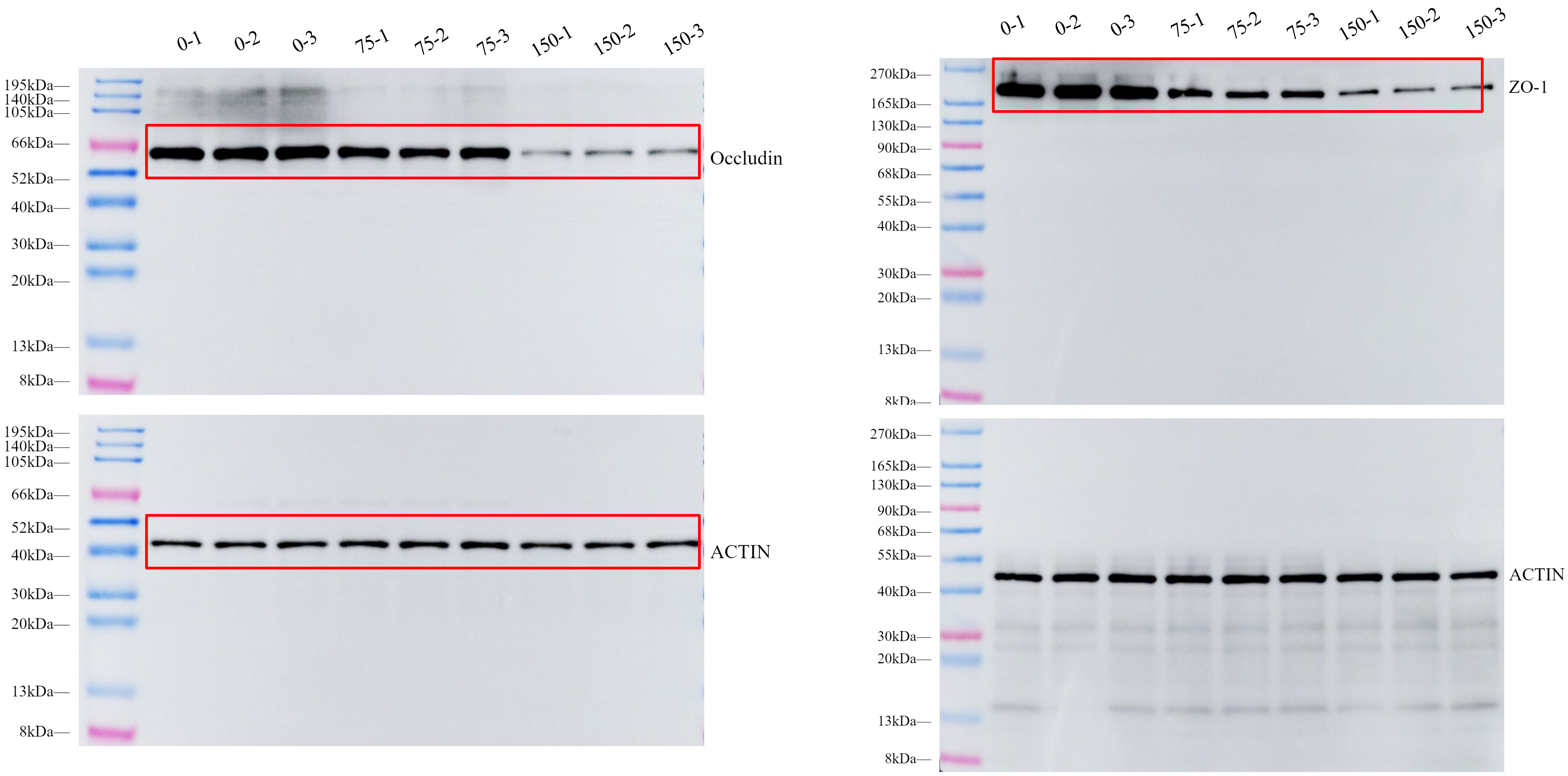
0-1
0-2
0-3
75-1
75-2
75-3
150-1
150-2
150-3
195kDa
140kDa
105kDa
66kDa
52kDa
40kDa
30kDa
20kDa
13kDa
8kDa
Occludin
ACTIN
270kDa
165kDa
130kDa
90kDa
68kDa
55kDa
ZO-1

# Fig. S6

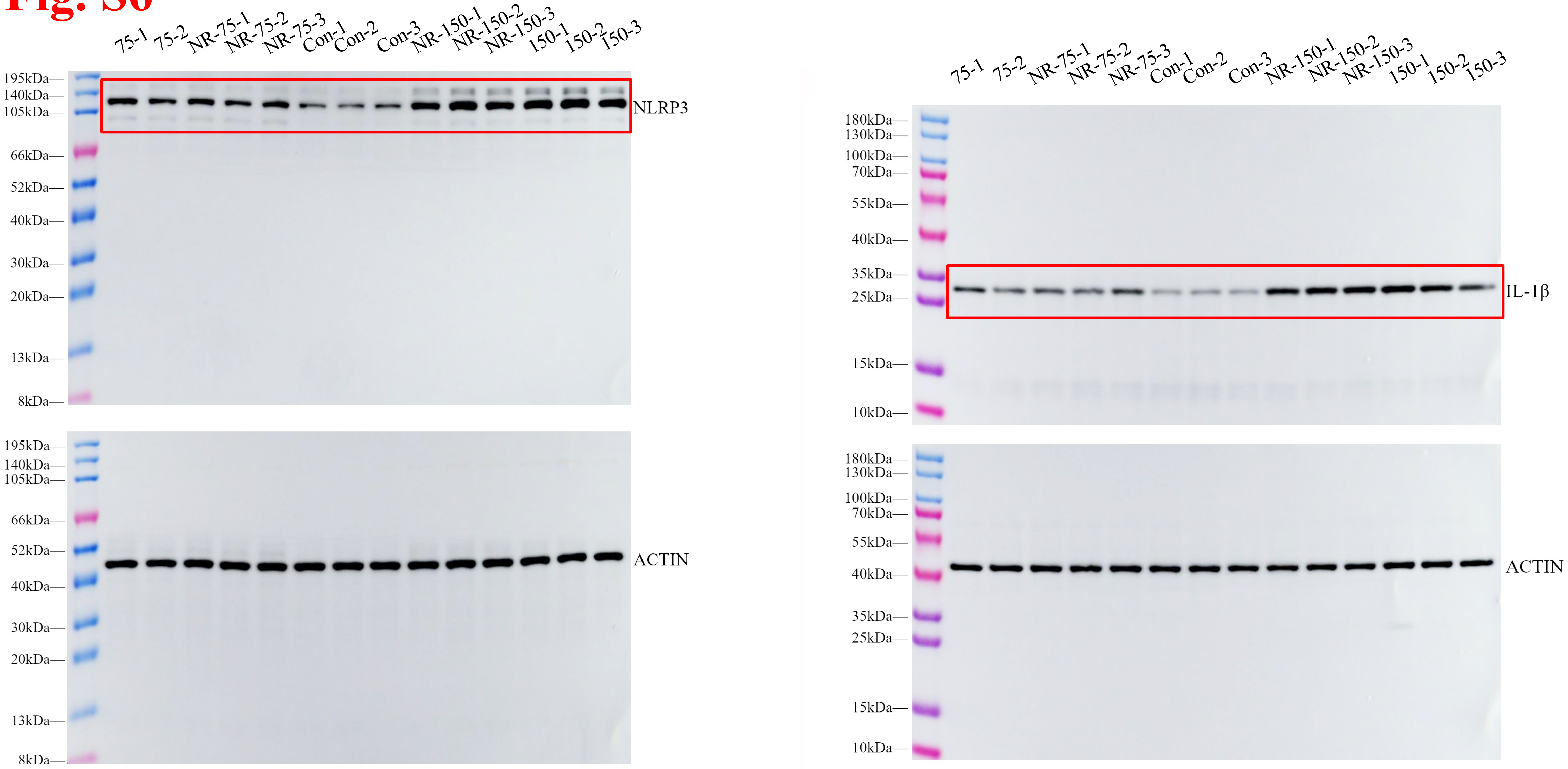
75-1
75-2
NR-75-1
NR-75-2
NR-75-3
Con-1
Con-2
Con-3
NR-150-1
NR-150-2
NR-150-3
150-1
150-2
150-3
195kDa
140kDa
105kDa
66kDa
52kDa
40kDa
30kDa
20kDa
13kDa
8kDa
NLRP3
ACTIN
180kDa
130kDa
100kDa
70kDa
55kDa
35kDa
25kDa
15kDa
10kDa
IL-1β

Fig. S6

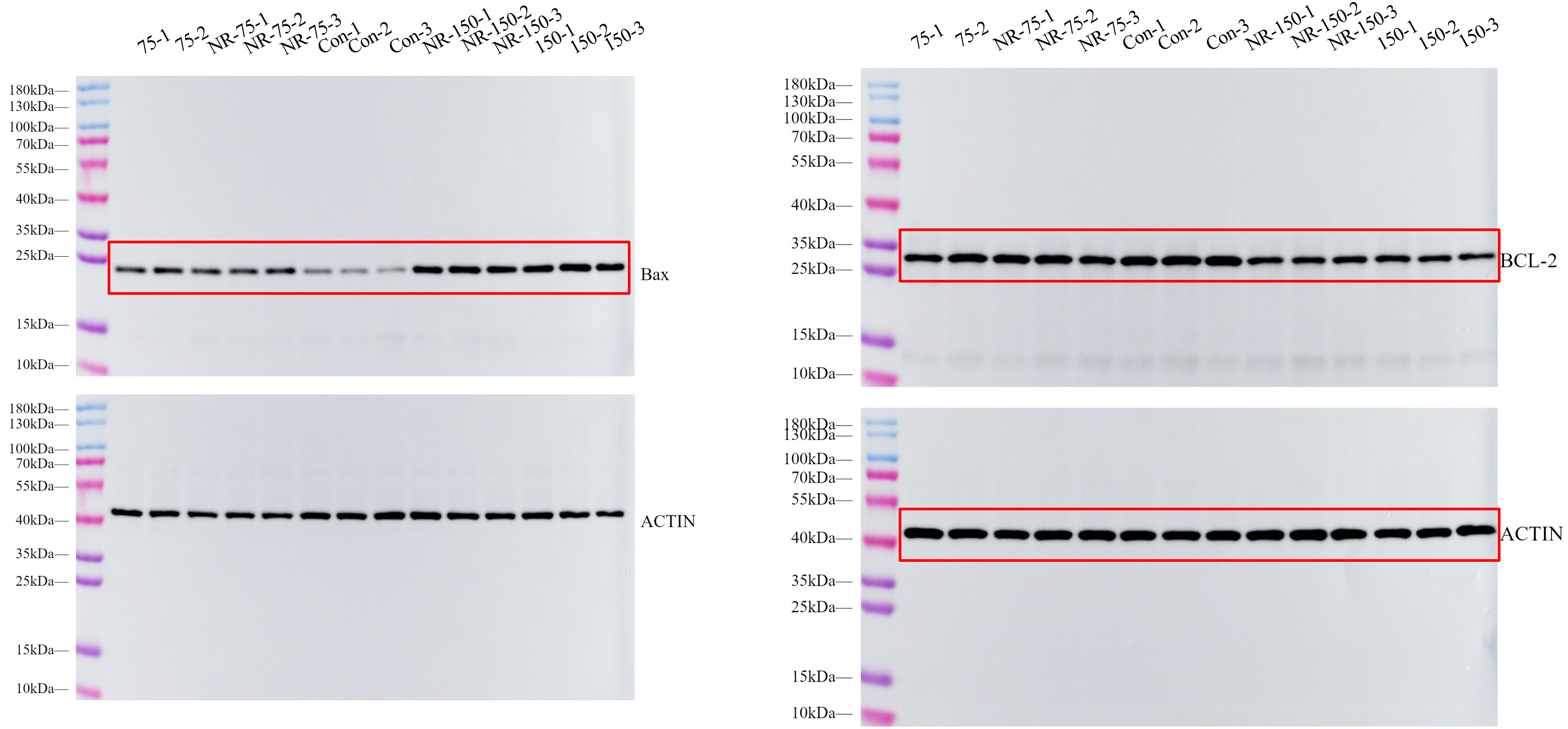
75-1
75-2
NR-75-1
NR-75-2
NR-75-3
Con-1
Con-2
Con-3
NR-150-1
NR-150-2
NR-150-3
150-1
150-2
150-3
180kDa
130kDa
100kDa
70kDa
55kDa
40kDa
35kDa
25kDa
15kDa
10kDa
Bax
ACTIN
BCL-2
ACTIN

# Fig. S7

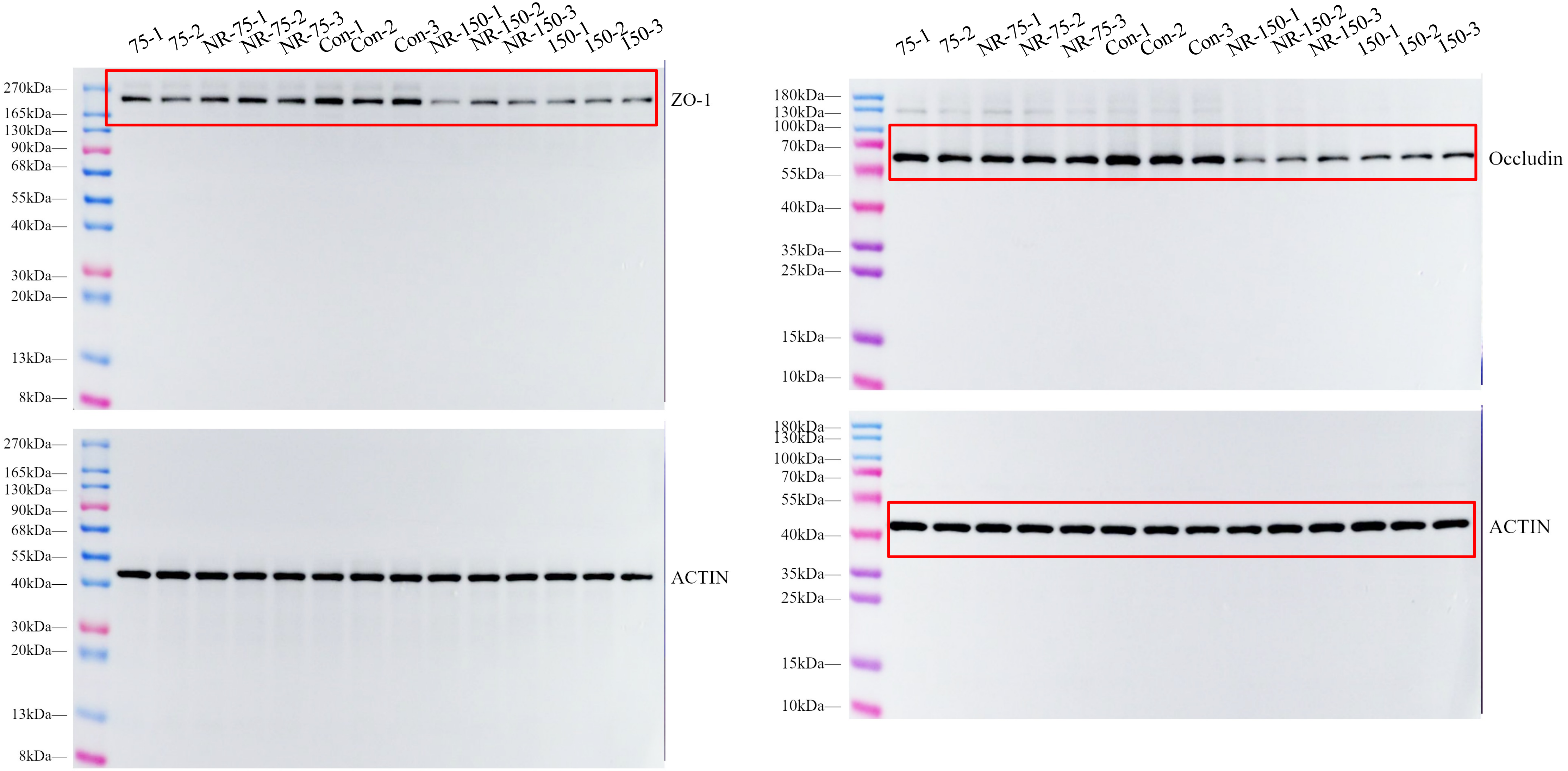
75-1
75-2
NR-75-1
NR-75-2
NR-75-3
Con-1
Con-2
Con-3
NR-150-1
NR-150-2
NR-150-3
150-1
150-2
150-3
270kDa
165kDa
130kDa
90kDa
68kDa
55kDa
40kDa
30kDa
20kDa
13kDa
8kDa
ZO-1
ACTIN
180kDa
130kDa
100kDa
70kDa
55kDa
40kDa
35kDa
25kDa
15kDa
10kDa
Occludin
ACTIN

# Fig. 6

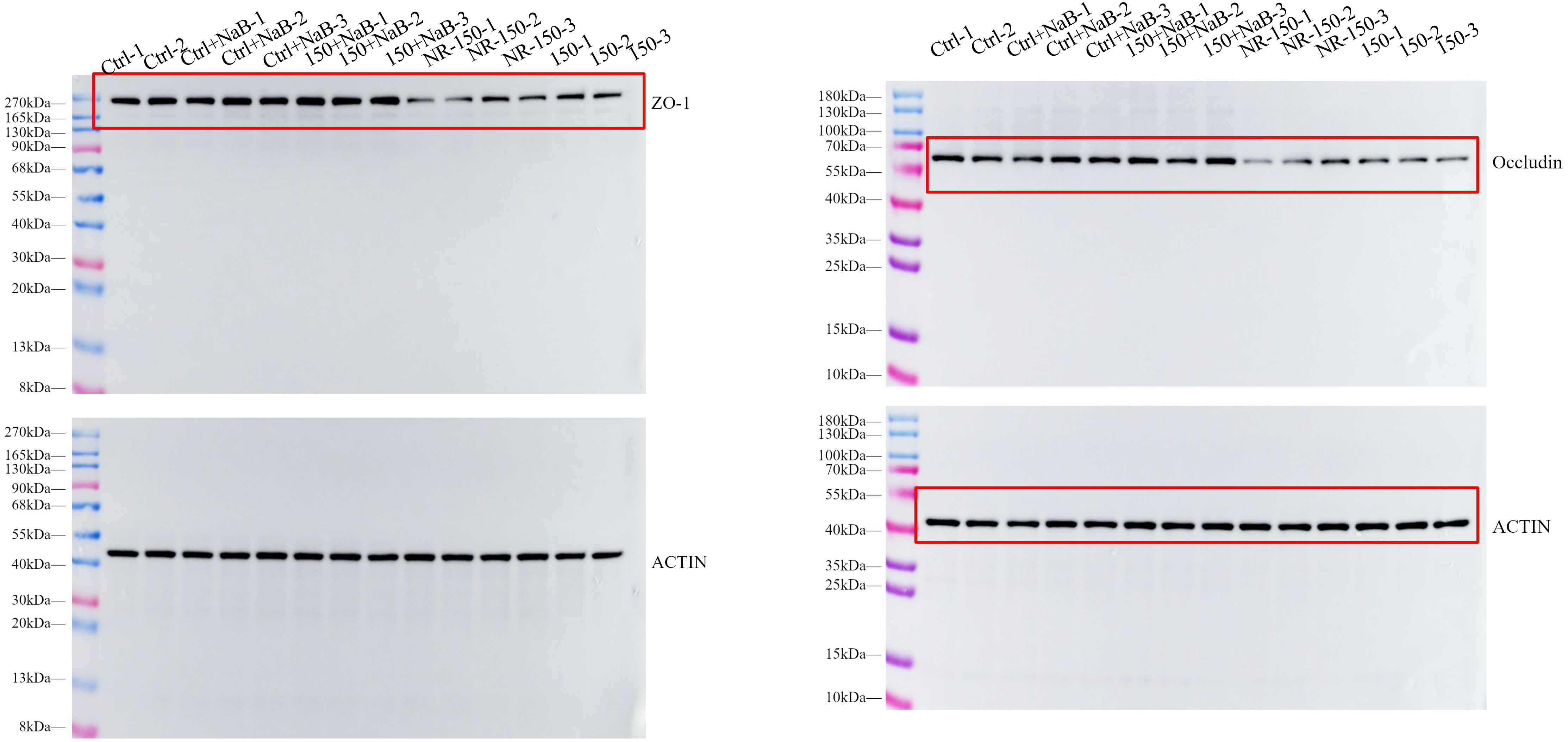

Ctrl-1
Ctrl-2
Ctrl+NaB-1
Ctrl+NaB-2
Ctrl+NaB-3
150+NaB-1
150+NaB-2
150+NaB-3
NR-150-1
NR-150-2
NR-150-3
150-1
150-2
150-3
270kDa
165kDa
130kDa
90kDa
68kDa
55kDa
40kDa
30kDa
20kDa
13kDa
8kDa
ZO-1
ACTIN
180kDa
130kDa
100kDa
70kDa
55kDa
40kDa
35kDa
25kDa
15kDa
10kDa
Occludin
ACTIN

# Fig. 7

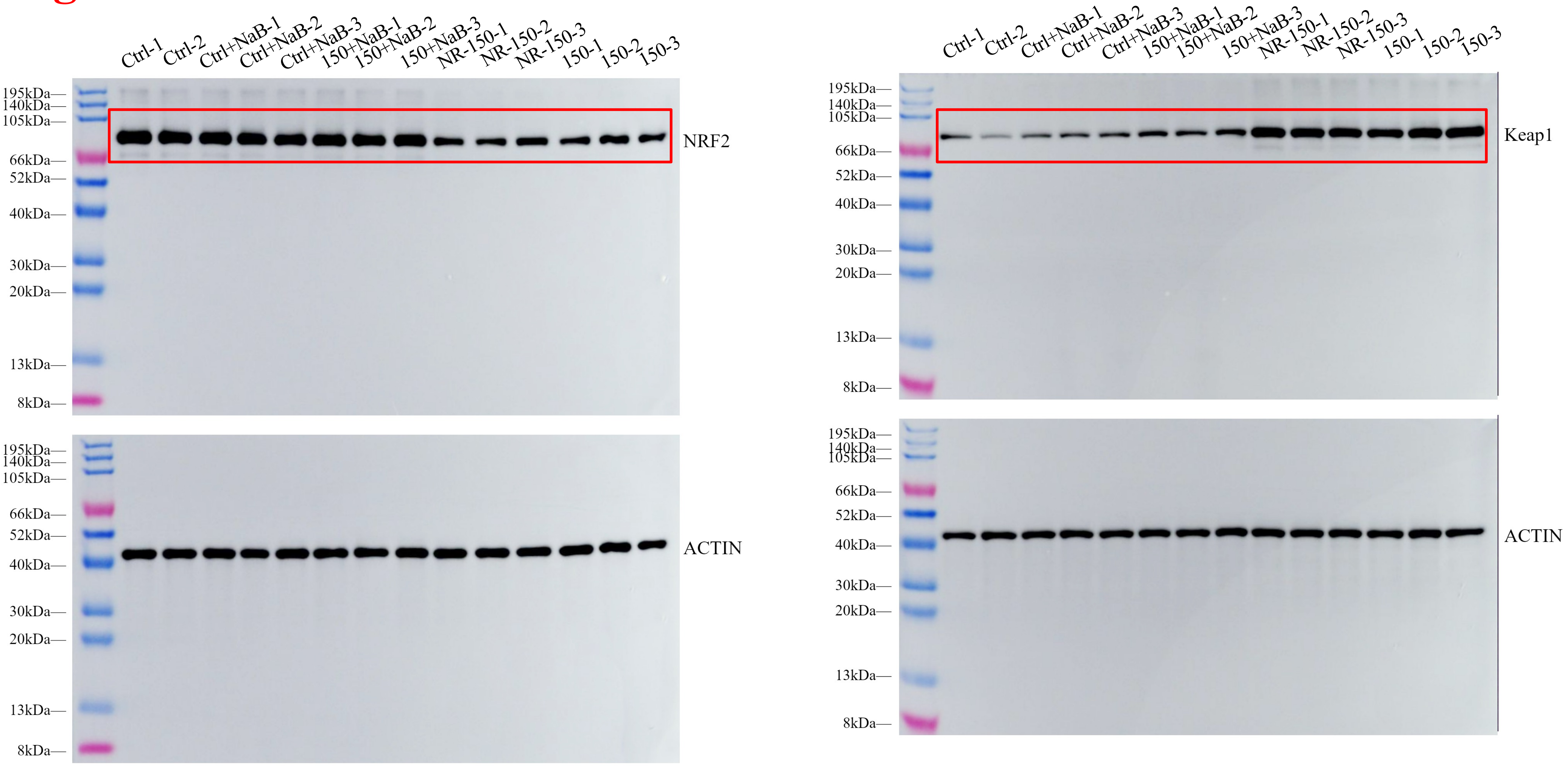
Ctrl-1
Ctrl-2
Ctrl+NaB-1
Ctrl+NaB-2
Ctrl+NaB-3
150+NaB-1
150+NaB-2
150+NaB-3
NR-150-1
NR-150-2
NR-150-3
150-1
150-2
150-3
195kDa
140kDa
105kDa
66kDa
52kDa
40kDa
30kDa
20kDa
13kDa
8kDa
NRF2
ACTIN
Keap1
ACTIN

# Fig. 7

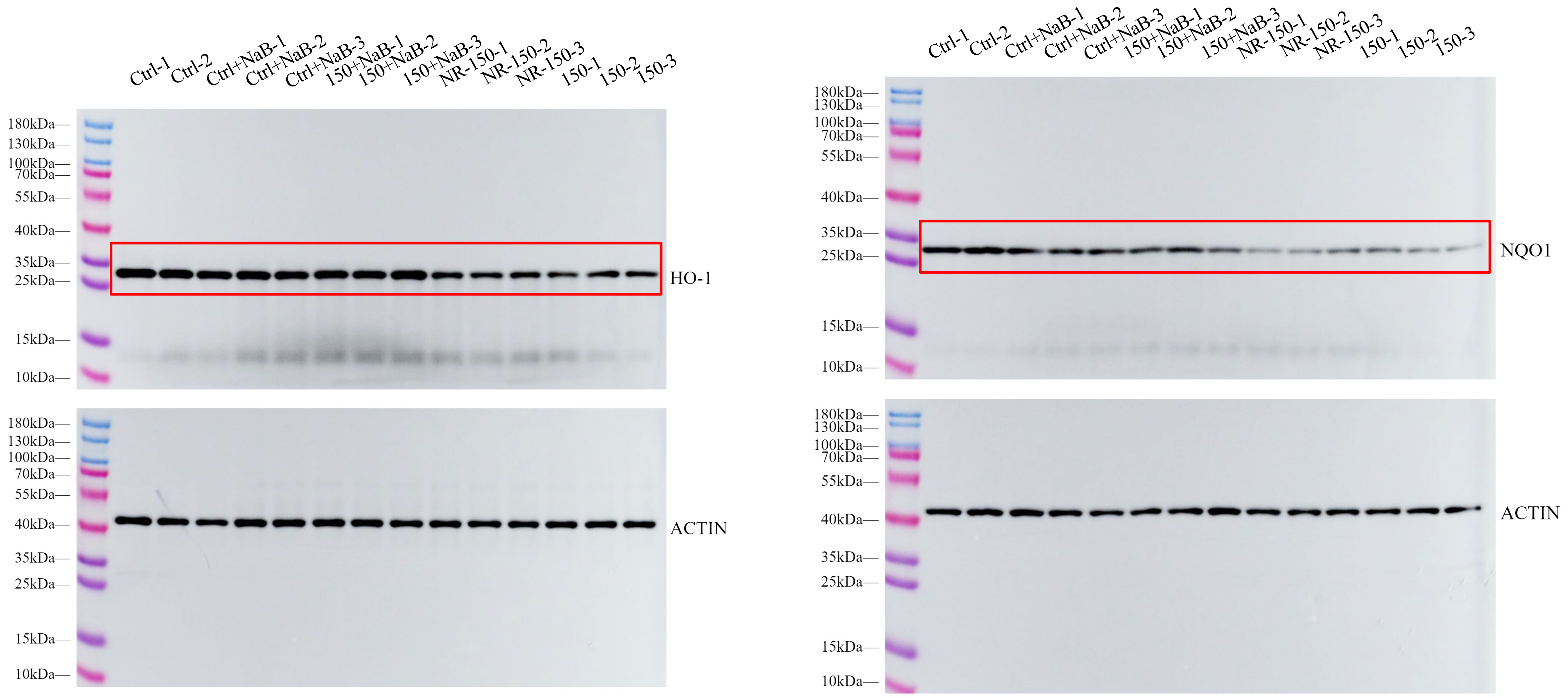
Ctrl-1
Ctrl-2
Ctrl+NaB-1
Ctrl+NaB-2
Ctrl+NaB-3
150+NaB-1
150+NaB-2
150+NaB-3
NR-150-1
NR-150-2
NR-150-3
150-1
150-2
150-3
180kDa
130kDa
100kDa
70kDa
55kDa
40kDa
35kDa
25kDa
15kDa
10kDa
HO-1
ACTIN
NQO1
ACTIN

# Fig. 7

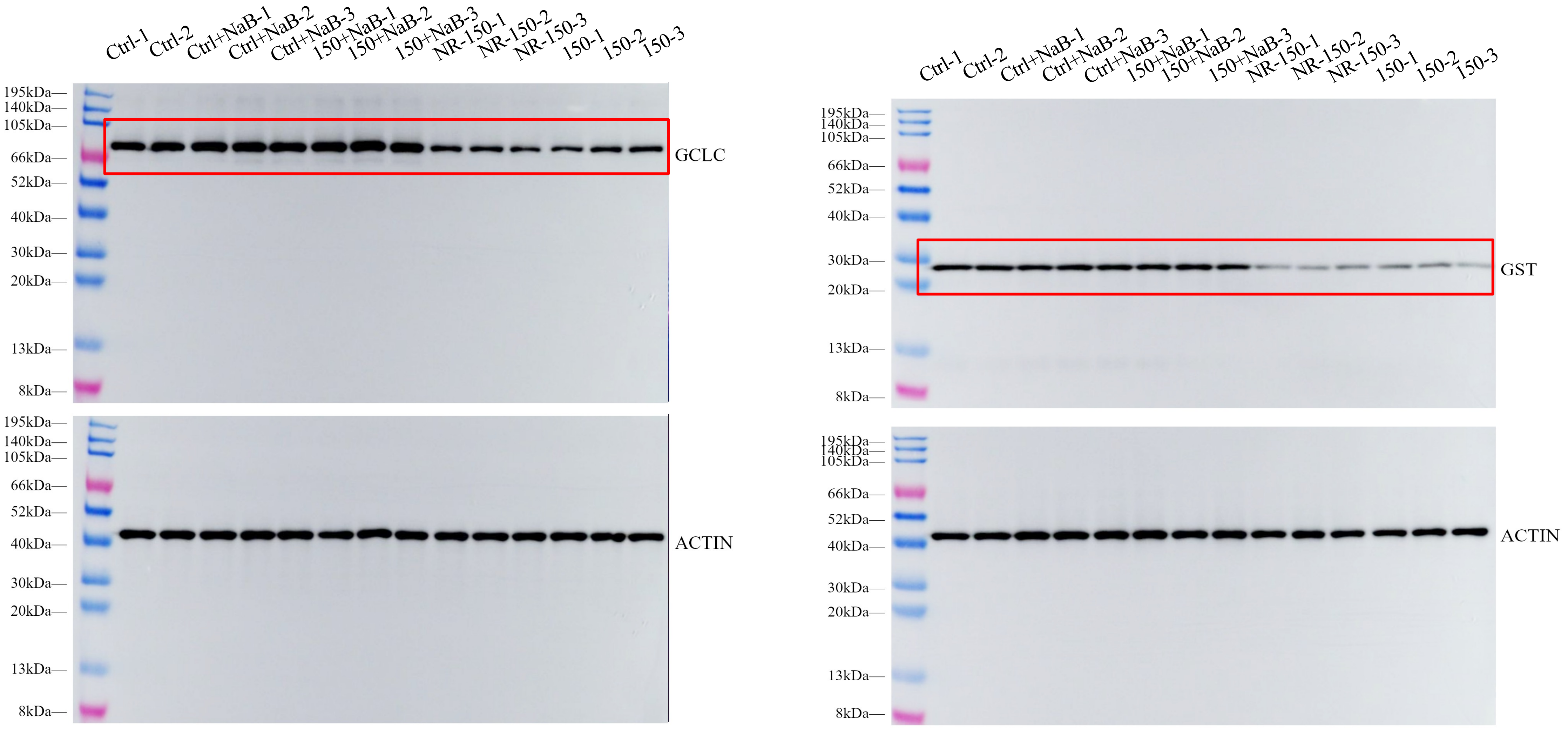
Ctrl-1
Ctrl-2
Ctrl+NaB-1
Ctrl+NaB-2
Ctrl+NaB-3
150+NaB-1
150+NaB-2
150+NaB-3
NR-150-1
NR-150-2
NR-150-3
150-1
150-2
150-3
195kDa
140kDa
105kDa
66kDa
52kDa
40kDa
30kDa
20kDa
13kDa
8kDa
GCLC
ACTIN
GST
ACTIN

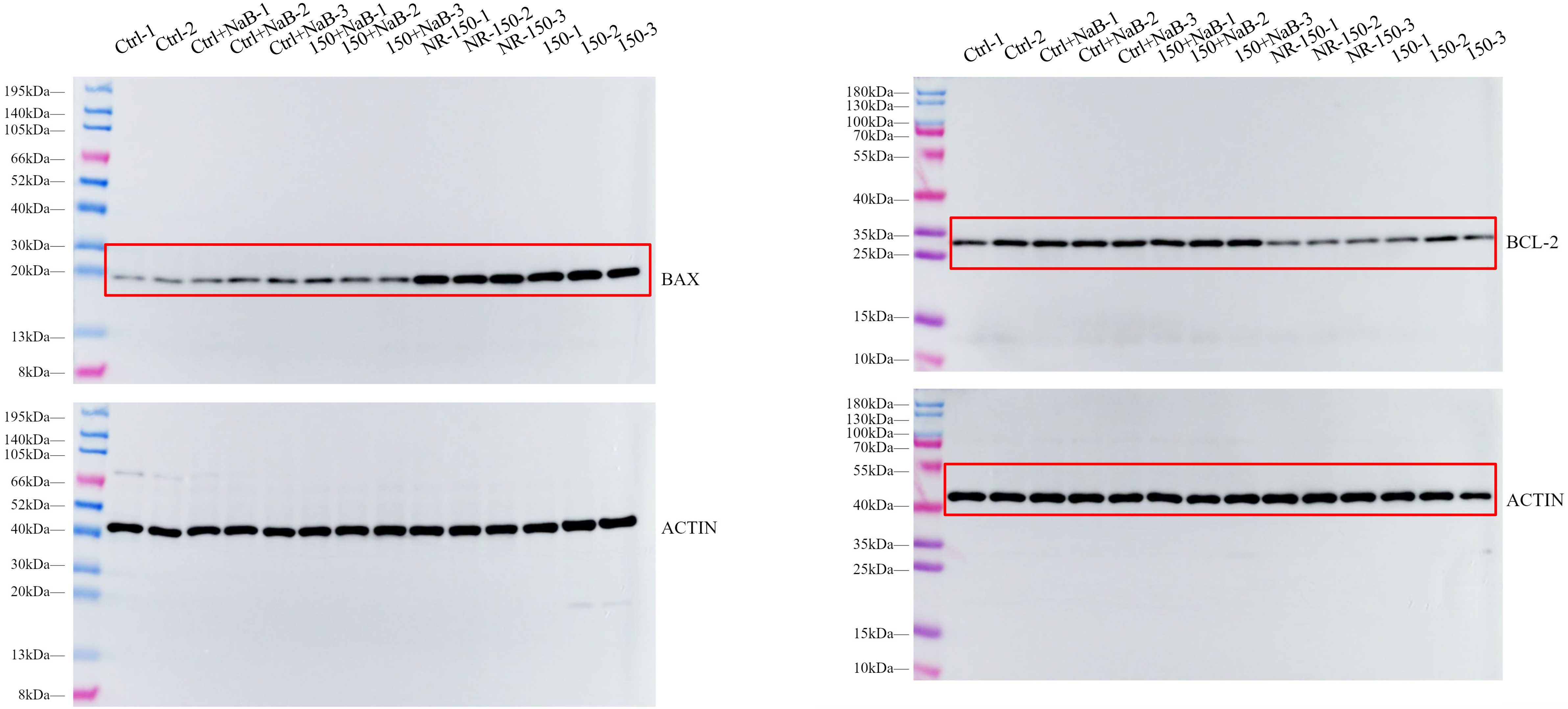
Ctrl-1
Ctrl-2
Ctrl+NaB-1
Ctrl+NaB-2
Ctrl+NaB-3
150+NaB-1
150+NaB-2
150+NaB-3
NR-150-1
NR-150-2
NR-150-3
150-1
150-2
150-3
195kDa
140kDa
105kDa
66kDa
52kDa
40kDa
30kDa
20kDa
13kDa
8kDa
BAX
ACTIN
180kDa
130kDa
100kDa
70kDa
55kDa
35kDa
25kDa
15kDa
10kDa
BCL-2

# Fig. 7
