# Supplementary material for: Polystyrene microplastics impair sheep spermatogenesis by disrupting rumen microbiota-derived butyrate signaling
Source: J Anim Sci Biotechnol. 2026 Sep 9;17:177. doi: 10.1186/s40104-026-01490-z (PMC13555869; doi:10.1186/s40104-026-01490-z)
Supplement: Supplementary file 2 [file 40104_2026_1490_MOESM1_ESM.docx]

**Supplementary information**

**Polystyrene microplastics impair sheep spermatogenesis by disrupting rumen microbiota-derived butyrate signaling**

Juanjuan Song^1,2^, Keyan Ma^1,2^, Xingcai Qi^1,2^, Dengpan Li^1,2^, Xingxu Zhao^2^, Youji Ma^1,2*^

^1^College of Animal Science and Technology, Gansu Agricultural University, Lanzhou 730070, China

^2^Gansu Key Laboratory of Animal Generational Physiology and Reproductive Regulation, Lanzhou 730070, China

*Corresponding author: (Youji Ma, email: [myj1124@1](mailto:myj1124@1)63.com)

**Supplementary Methods 1.1 Dose Rationale**

Microplastic contamination has been reported in ruminant feedstuffs. A recent survey of dairy farms in northwestern Italy reported microplastic abundances of 37.9 ± 7.94 items/g in corn silage, 16.4 ± 3.81 items/g in hay, and 16.3 ± 3.76 items/g in high-protein feedstuff [2]. In that study, fiber-shaped particles were the dominant morphology, and particle lengths ranged from 0.030 to 5 mm [2]. Based on fiber mass estimates (0.1-1 μg per fiber) adopted in international microplastic risk assessment frameworks [12], the reported particle abundances correspond to 3.79–37.9 mg MPs/kg feed for corn silage, 1.64–16.4 mg MPs/kg feed for hay, and 1.63–16.3 mg MPs/kg feed for high-protein feedstuff.

Against this environmental exposure background, 75 mg PS-MPs/kg diet was selected as an environmentally informed high-end dietary exposure level. This dose exceeds the mass range estimated from individual feed components, but was chosen to account for mixed-ration feeding under intensive farming conditions. The higher dose of 150 mg/kg diet was included as an elevated exposure level to examine dose–response effects and adverse outcomes under higher exposure conditions. Thus, the two doses should be interpreted as high-end and elevated controlled dietary exposure levels, rather than precise reproductions of typical environmental exposure.

In addition, because corn silage has been identified as a major contributor to dietary microplastic contamination, imported alfalfa hay and soybean meal were selected as the primary dietary ingredients to reduce background microplastic interference and improve experimental precision.

**Supplementary Methods 1.2 Endotoxin detection of PS-MPs**

Polystyrene microplastic (PS-MPs) particles were prepared as a 1 mg/mL suspension in ultrapure water (Milli-Q, 18.2 MΩ·cm), followed by vortexing for 10 min and ultrasonic treatment in a water bath for 10 min to ensure uniform dispersion. Endotoxin content was determined using a chromogenic Limulus Amebocyte Lysate (LAL) assay kit (Associates of Cape Cod, USA). The assay was performed according to the manufacturer’s instructions: the microplastic suspensions were added to endotoxin-free 96-well plates, with both negative and positive controls included. All samples were measured colorimetrically, and the endotoxin concentrations were expressed in EU/mL. The threshold criterion was defined as follows: if the lipopolysaccharide (LPS) concentration in the microplastic suspension was below 0.05 EU/mL, the particles were considered free of endotoxin contamination and suitable for subsequent animal exposure experiments.

**Supplementary Methods 1.3 Untargeted metabolomics**

The collected samples were first extracted using a mixture of methanol and acetonitrile, and the resulting supernatants were analyzed by LC–MS. Chromatographic separation was performed on an HSS T3 column (Waters, USA) mounted on a Vanquish Flex UHPLC platform (Thermo, USA). The injection volume was set to 2 μL, with a column temperature of 40 °C and a flow rate maintained at 0.3 mL/min. The mobile phase system included four solvents: (A) water with 0.1% formic acid, (B) methanol containing 0.1% formic acid, (C) water with 0.05% acetic acid, and (D) methanol containing 0.05% acetic acid. Following chromatographic separation, mass spectrometric detection was carried out on a Q Exactive HF-X instrument (Thermo, USA) equipped with an electrospray ionization (ESI) source and operated in both positive and negative ion modes. The major MS settings were: sheath gas 50, auxiliary gas 13, spray voltage ± 2.5 kV, S-Lens RF level 50, capillary temperature 325 °C, auxiliary gas temperature 300 °C, normalized collision energy (NCE) 30, and Top-N acquisition of 10. The scan range covered m/z 70–1050. Raw LC–MS files were processed with Compound Discoverer 3.0 to perform peak alignment, correction of retention time drift, and signal abundance extraction. Metabolite annotation relied on accurate mass information together with MS/MS spectral matching. Subsequent analyses, including dimensionality reduction, differential metabolite screening, and pathway enrichment, were performed using MetaboAnalyst 6.0 ([www.metaboanalyst.ca](http://www.metaboanalyst.ca)). The robustness of the OPLS-DA models was evaluated using permutation tests (*n* = 200). Model validity was confirmed by ensuring that the permuted Q² values were lower than those of the original model and that the regression line of Q² had a negative intercept. Differential metabolites were identified based on a combination of VIP > 1.0 and adjusted P values (false discovery rate, FDR < 0.05).

**Supplementary Methods 1.4 Single cell RNA-sequencing and analysis**

Single-cell libraries were generated using the 10× Genomics Chromium Single Cell 3′ Library & Gel Bead Kit v2 following the manufacturer’s protocol. Fresh testicular tissues were washed, minced, and digested with collagenase IV (1 mg/mL, 37°C, 15 min). The digestion was quenched with DMEM (10% FBS), filtered through a 40 μm strainer, and pelleted. Cells were washed in PBS containing 0.04% BSA and assessed for viability using trypan blue (>80%). A suspension of 1,000 cells/μL was loaded onto the Chromium Single Cell System to generate GEMs for barcoding and cDNA library construction. Sequencing was performed on an Illumina NovaSeq 6000 (PE150).

Raw data were processed with CellRanger v3.0.2 using the 10× Genomics pre-built sheep genome (Oar_rambouillet_v1.0). Downstream clustering, quality control, and cluster marker identification (“FindAllMarkers”) were conducted in Seurat v3.1.5. Cell clusters were annotated based on canonical marker genes. Differentially expressed genes were subjected to functional enrichment analysis using Metascape. Single-cell pseudotime trajectories were reconstructed with Monocle 2 (v2.16.0) following the official guidelines. For lineage-specific trajectory analysis, relevant cell subsets were extracted from the Seurat object, and Monocle objects were generated using “newCellDataSet”.

**Supplementary Methods 1.5 Py-GC/MS**

Before analyzing microplastics in testicular tissue samples using Py-GC/MS, we digested the tissue according to established methods [25]. Briefly, 68% concentrated nitric acid was added to the testicular samples at a ratio of 10 mL acid per 1.0 g of tissue. The samples were then placed on a shaker (MaxQ™ 4000, Thermo Scientific, USA) and continuously agitated at 120 rpm for 72 h at room temperature (25 °C) until complete tissue dissolution. After digestion, filtered Milli-Q® ultrapure water (0.22 µm) was added to the sample bottles for subsequent analysis.

The samples were quantitatively analyzed for six common microplastics, including polyvinyl chloride (PVC), polypropylene (PP), polystyrene (PS), polyethylene (PE), polyamide-66 (PA-66), and polyethylene terephthalate (PET), using pyrolysis-gas chromatography-mass spectrometry (Py-GC/MS, Frontier Py-3030D; Shimadzu GCMS-QP 2020, Japan). The pyrolysis temperature was set to 550 °C, with high-purity helium (5.0 grade) as the carrier gas at a constant flow rate of 1 mL min⁻¹. The chromatographic separation was achieved using an Rtx-5MS column (30 m × 0.25 mm × 0.25 μm, Shimadzu, Japan). The temperature program started at 40 °C for 2 minutes, then increased at a rate of 20 °C min⁻¹ to 320 °C, where it was maintained for 14 minutes. Selective ion monitoring (SIM) mode was employed for both qualitative and quantitative analysis of the target polymer’s characteristic fragments, with a scanning range of m/z 29–600. Calibration curves were established using standard microplastic samples, as detailed in Table S4, to determine the microplastic content in the testicular tissue. The microplastic concentration was normalized to the weight of the testicular samples and the detected microplastic levels in the samples [26].

**Supplementary Methods 1.6 Metagenomics**

Metagenomic DNA was extracted from rumen fluid samples using the QIAamp PowerFecal Pro DNA Kit. Following quality assessment with a NanoDrop One spectrophotometer and Qubit 4.0 fluorometer, sequencing libraries were prepared with the Illumina DNA Prep Kit. The libraries were subjected to paired-end (2×150 bp) shotgun sequencing on an Illumina NovaSeq 6000 platform, yielding ≥10 Gb of raw data per sample. Bioinformatics analysis included the following steps: (1) raw read quality control and adapter trimming using Fastp (v0.23.2); (2) co-assembly of high-quality reads with MEGAHIT (v1.2.9) and open reading frame prediction via MetaGeneMark (v3.38); (3) construction of a non-redundant gene catalog using CD-HIT (v4.8.1) at 95% amino acid identity; (4) gene abundance quantification by mapping reads back to the gene catalog with Bowtie2, followed by functional annotation against the NR, KEGG, and CAZy databases using DIAMOND; (5) differential functional analyses were performed based on KEGG pathway abundances. P-values obtained from multiple comparisons were adjusted using the Benjamini–Hochberg false discovery rate (FDR) method, and pathways with FDR-adjusted P values < 0.05 were considered statistically significant; (6) microbial diversity analysis based on the gene abundance matrix, including α-diversity (Shannon index) and β-diversity (based on binary Jaccard distances) with statistical significance of group differences assessed by ANOSIM.

**Supplementary Figure**

**Fig. S1** Characterization of PS-MPs. **A** FTIR spectrum of PS-MPs. **B** SEM image of PS-MPs (scale bar = 100 μm). **C** Particle size distribution of PS-MPs. **D** Endotoxin levels in the PS-MPs suspension. The dashed line indicates the endotoxin contamination threshold (0.05 EU/mL).

**Fig. S2** Marker gene expression and validation of spermatogenic subpopulations in sheep testes. **A** Schematic workflow of single-cell RNA sequencing and downstream bioinformatic analysis. **B** Dot plot displaying canonical marker gene expression across identified testicular cell clusters. Dot size represents the percentage of expressing cells, and color intensity indicates average expression level. **C–F** Violin plots showing expression patterns of stage-specific marker genes across spermatogonia.

**Fig. S3** Py-GC/MS chromatograms.

**Fig. S4** Effects of PS-MPs exposure on rumen microbial diversity and function. **A** PS-MPs exposure reduced rumen microbial α-diversity. Box-and-whisker plots illustrate the richness indices (Observed species, ACE, and Chao1) and diversity indices (Shannon, Simpson, and Pielou’s evenness) among groups. **B** Taxonomic composition at the phylum level. **C** Comparison of the Firmicutes-to-Bacteroidetes (F/B) ratio among groups (*P* = 0.001). **D** Taxonomic composition at the genus level. **E** LEfSe analysis and cladogram showing taxa differentially enriched among groups. **F** Analysis of KEGG functional composition of the microbiota across groups. *P*-values are shown in each panel, and statistical significance is indicated by asterisks (**P* < 0.05, ***P* < 0.01, ****P* < 0.001).

**Fig. S5 Systemic hormonal, inflammatory, and antioxidant disturbances failed to recover after cessation of exposure. A Schematic illustration of the exposure–natural recovery experimental design. B Body weight changes during the recovery phase. C Representative testicular gross morphology at the end of the recovery period. D Transverse and longitudinal testicular diameters. E Testicular weight. F Testis-to-body weight ratio (testis index). G Serum reproductive hormone levels, including FSH, INHB, T, LH, and FSH/LH ratio. H Serum inflammatory cytokine concentrations (IL-1β, IL-6, TNF-α, IL-4, and IL-10). I Serum antioxidant enzyme activities (GSH-Px, CAT, SOD, T-AOC) and MDA content.** Data are presented as mean ± SEM (*n* = 6). ns, not significant; **P* < 0.05; ***P* < 0.01; ****P* < 0.001.

**Fig. S6** PS-MPs–induced testicular histopathological damage, inflammation, and apoptosis failed to recover naturally. **A** H&E staining of testicular sections, scale bar = 50 μm. **B** Average diameter of seminiferous tubules. **C** Johnsen score. **D** TEM images, scale bar = 5 μm. **E** Masson’s trichrome staining, scale bar = 50 μm. **F** Percentage of Masson-positive area. **G** Immunofluorescence staining of NLRP3 (red) and IL-1β (green) with DAPI nuclear staining (blue) in testicular tissue (scale bar = 20 μm). **H–I** Quantification of IL-1β- and NLRP3-positive cells. J: TUNEL staining (scale bar = 50 μm). **K** Percentage of TUNEL-positive germ cells. **L–M** Representative western blot images and quantification of NLRP3, IL-1β, BAX, and BCL-2 protein expression in testicular tissue, with β-actin used as a loading control. Data are presented as mean ± SEM (*n* = 6). ns, not significant; **P* < 0.05; ***P* < 0.01; ****P* < 0.001.

**Fig. S7 Ruminal butyrate depletion and epithelial tissue damage persisted after natural recovery. A** Butyrate concentration in rumen fluid. **B** Serum β-hydroxybutyrate concentration. **C** Representative western blot images and quantification of ZO-1 and Occludin protein expression in rumen epithelium. **D** H&E staining of rumen (scale bar = 200 μm). Immunohistochemical staining of ZO-1 and Occludin in rumen epithelium (Scale bar = 100 μm). **E** Quantification of rumen papilla height, papilla width, and muscular thickness. **F** ZO-1 and Occludin-positive cells. Data are presented as mean ± SEM (*n* = 6). ns, not significant; **P* < 0.05; ***P* < 0.01; ****P* < 0.001.

**Fig. S8 NaB supplementation significantly restored testicular morphology and suppressed inflammation and apoptosis following PS-MPs exposure. A Representative gross morphology of testes from Ctrl, Ctrl+NaB, and 150+NaB groups. B Transverse and longitudinal testicular diameters. C Testicular weight. D Testis-to-body weight ratio (testis index). E Antioxidant enzyme activities, including SOD, CAT, GSH-Px, and T-AOC, and MDA content in testicular tissue homogenates. F Representative histological analyses of testicular tissue, including H&E staining (scale bar = 50 μm), TEM images (scale bar = 5 μm), Masson’s trichrome staining (scale bar = 50 μm), immunofluorescence staining of NLRP3 (red) and IL-1β (green) with DAPI nuclear counterstaining (blue) (scale bar = 20 μm), and TUNEL staining (scale bar = 50 μm). G Quantitative analysis of average seminiferous tubule diameter and Johnsen score. H Percentage of Masson-positive area. I Percentage of NLRP3- and IL-1β-positive cells. J Percentage of TUNEL-positive germ cells. Data are presented as mean ± SEM (*n* = 6). ns, not significant; **P* < 0.05; ***P* < 0.01; ****P* < 0.001.**

**Fig. S9** Histological and seminal examinations of cauda epididymis. **A** Representative H&E staining of the cauda epididymis (scale bar = 100 μm). **B** Sperm concentration in the cauda epididymis. **C** Representative semen smears stained with Wright–Giemsa staining (scale bar = 20 μm). **D** Percentage of morphologically abnormal sperm, including head–tail separation, coiled tail, and broken tail (≥200 sperm counted per sample, *n* = 6). Data are presented as mean ± SEM. ns, not significant; **P* < 0.05; ***P* < 0.01; ****P* < 0.001.

**Fig. S10 Quantification of Nrf2 pathway and apoptosis-related proteins in testicular tissue. A** Percentages of positive cells for Nrf2, Keap1, HO-1, NQO1, GCLC, and GST assessed by immunofluorescence. **B** Relative protein expression levels of Nrf2, Keap1, HO-1, NQO1, GCLC, GST, BAX, and BCL-2 in testicular tissue. Data are presented as mean ± SEM (*n* = 6). ns, not significant; **P* < 0.05; ***P* < 0.01; ****P* < 0.001.

**Supplementary Table**

**Table S1** Mapping of reported ruminant feed microplastic contamination to approximate mass-based estimates and experimental PS-MPs doses

| **Source** | **Reported particle abundance** | **Converted particle abundance** | **Assumed particle mass for approximate conversion** | **Estimated mass-based contamination** |
| --- | --- | --- | --- | --- |
| Corn silage from dairy farms in northwestern Italy [2] | 37.9 particles/g | 37,900 particles/kg feed | 0.1–1 μg/particle | 3.79–37.9 mg MPs/kg feed |
| Hay from dairy farms in northwestern Italy [2] | 16.4 particles/g | 16,400 particles/kg feed | 0.1–1 μg/particle | 1.64–16.4 mg MPs/kg feed |
| High-protein feedstuff from dairy farms in northwestern Italy [2] | 16.3 particles/g | 16,300 particles/kg feed | 0.1–1 μg/particle | 1.63–16.3 mg MPs/kg feed |
| Total | | | | 7.06–70.6 mg MPs/kg feed |
| Present study: low exposure level |  |  |  | 75 mg PS-MPs/kg diet |
| Present study: high exposure level |  |  |  | 150 mg PS-MPs/kg diet |

**Table S2** Kit information

| **kit** | **Cat. No.** | **kit** | **Cat. No.** | **kit** | **Cat. No.** |
| --- | --- | --- | --- | --- | --- |
| FSH | H101 | IL-1β | H002 | T-AOC | A015 |
| LH | H206 | IL-4 | H005 | SOD | A001 |
| T | H090 | IL-6 | H007 | CAT | A007 |
| INHB | H320 | IL-10 | H009 | GSH-Px | A005 |
| BHB | E030 | TNF-α | H052 | MDA | A003 |

**Table S3** Primary and secondary antibody information

| **Primary antibody** | **Manufacturer** | **Catalog No.** | **Dilution ration** | |
| --- | --- | --- | --- | --- |
|  |  |  | **WB** | **IHC/IF** |
| NLRP3 | Abmart | P60622R3 | 1:1500 | 1:300 |
| IL-1β | Abmart | PC6319 | 1:1000 | 1:200 |
| BAX | Abmart | T40051 | 1:1000 | 1:500 |
| BCL-2 | Abmart | T40056 | 1:1000 | 1:500 |
| ZO-1 | Thermo Fisher | PA5-28858 | 1:2000 | 1:300 |
| Occludin | Thermo Fisher | 40-4700 | 1:1000 | 1:300 |
| HO-1 | Thermo Fisher | PA5-7783 | 1:1000 | 1:300 |
| NQO1 | Thermo Fisher | PA5-19624 | 1:1000 | 1:300 |
| Nrf2 | Abmart | T55136S | 1:1000 | 1:300 |
| Keap1 | Bioss | bs-4900R | 1:500 | 1:200 |
| GST | Abcam | ab233112 | 1:1000 | 1:300 |
| GCLC | Thermo Fisher | Pa5-103134 | 1:2000 | 1:500 |
| β-actin | Thermo Fisher | MA5-11869 | 1:1000 | - |
| Secondary antibody |  |  |  |  |
| Goat anti-Rabbit IgG HRP | Thermo Fisher | 31460 | 1:10,000 | 1:800 |
| Goat anti-Mouse IgG HRP | Thermo Fisher | G-21040 | 1:15,000 | - |
| Alexa Fluor™ 555 | Thermo Fisher | A-21428 | - | 1:2000 |
| Alexa Fluor™ 488 | Thermo Fisher | A-11034 | - | 1:2000 |

**Table S4** Py-GC/MS calibration curves of polymers

| **Polymer** | **Characteristic compound** | **Retention time (min)** | **Calibration curve equation** | **R^2^** | **LOD**  **(μg)** | **LOQ**  **(μg)** |
| --- | --- | --- | --- | --- | --- | --- |
| PVC | naphthalene | 3.7 | y=24442.66x+3702.01 | 0.9981 | 0.02 | 0.06 |
| PP | 2,4-dimethyl-1-heptene | 5.5 | y =271533.00x-192346.98 | 0.9981 | 0.05 | 0.15 |
| PS | styrene dimer | 6.3 | y =233257.99x+355128.62 | 0.9945 | 0.02 | 0.06 |
| PE | n-monoene | 7.5 | y=20866.10x-5309.36 | 0.9965 | 0.02 | 0.06 |
| PA-66 | cyclopentanone | 10.8 | y=10455.52x-10508.99 | 0.9986 | 0.02 | 0.06 |
| PET | benzoic acid | 13.5 | y=26279.45x-5887.53 | 0.9986 | 0.02 | 0.06 |

Abbreviations: LOD, limit of detection; LOQ, limit of quantitation; PVC, polyvinyl chloride; PP, polypropylene; PS, polystyrene; PE, polyethylene; PA, polyamide; PET, polyethylene terephthalate.

**Table S5** Primer sequences

| **Genes** | **Forward sequences** | **Reverse sequences** | **Gene ID** |
| --- | --- | --- | --- |
| *Nrf2* | CCAAAGTGGACCGAACG | CCACCACCTCTTGATGATTG | [XM_015093345.4](https://www.ncbi.nlm.nih.gov/nuccore/XM_015093345.4) |
| *Keap1* | GACCCAGACACAGACACCTG | ACGAGTTCCCACTCTAGCCT | [XM_027969637.3](https://www.ncbi.nlm.nih.gov/nuccore/XM_027969637.3) |
| *HO-1* | GCCTTCTTCACTTTCCCCAATA | GGGTCATCTCCAGCGTGTTC | [NM_001317450.2](https://www.ncbi.nlm.nih.gov/nuccore/NM_001317450.2) |
| *NQO1* | CTGGAAGCTGCAGACCTTGT | GAGCCCTTTTGAGCCTAATCA | [XM_004015102.6](https://www.ncbi.nlm.nih.gov/nuccore/XM_004015102.6) |
| *GCLC* | GCGAGAAAGTGGCCATCAAC | AGTTCTCGAAGTCCGTCAGC | [XM_015102643.4](https://www.ncbi.nlm.nih.gov/nuccore/XM_015102643.4) |
| *GST* | GACCAGACCATTCTCAACTAC | AGCAGGATCATTTCACCCA | [NM_001114766.1](https://www.ncbi.nlm.nih.gov/nuccore/NM_001114766.1) |
| *β-actin* | AGCCTTCCTTCCTGGGCATGGA | GGACAGCACCGTGTTGGCGTAGA | [XM_060405599.1](https://www.ncbi.nlm.nih.gov/nuccore/XM_060405599.1) |

**Table S6** Microplastics profiles in testicular samples

| **Group** | **Samples**  **No.** | **Weight (g)** | **Total (g)** | **Microplastic content (μg/g)** | | | | | |
| --- | --- | --- | --- | --- | --- | --- | --- | --- | --- |
|  |  |  |  | **PVC** | **PP** | **PS** | **PE** | **PA66** | **PET** |
| 0 mg | 1 | 1.9363 | 0.3019 | - | 0.0308 | - | 0.1098 | 0.1613 | - |
|  | 2 | 1.5033 | 0.6126 | - | - | - | 0.3254 | 0.2867 | - |
|  | 3 | 1.6456 | 0.6081 | - | - | - | 0.1862 | 0.4219 | - |
|  | 4 | 1.2789 | 0.5167 | - | - | - | 0.1387 | 0.3780 | - |
|  | 5 | 1.7321 | 0.6796 | - | - | - | 0.2535 | 0.4261 | - |
|  | 6 | 1.8578 | 1.4329 | - | - | - | 0.4620 | 0.9709 | - |
| 75 mg | 7 | 1.8039 | 0.5251 | - | - | - | 0.1849 | 0.3402 | - |
|  | 8 | 1.4829 | 0.5149 | - | - | - | 0.1438 | 0.3711 | - |
|  | 9 | 1.3751 | 0.7254 | - | - | - | 0.3167 | 0.4078 | - |
|  | 10 | 1.7981 | 0.9277 | - | - | - | 0.2562 | 0.6715 | - |
|  | 11 | 1.4633 | 0.7754 | - | - | - | 0.2001 | 0.5753 | - |
|  | 12 | 1.5232 | 0.4006 | - | - | - | 0.1731 | 0.2275 | - |
| 150 mg | 13 | 1.6010 | 0.7740 | - | - | - | 0.2749 | 0.4991 | - |
|  | 14 | 1.8124 | 0.6404 | - | - | - | 0.1926 | 0.4478 | - |
|  | 15 | 1.5423 | 0.4091 | - | - | - | 0.1252 | 0.2839 | - |
|  | 16 | 1.5835 | 0.5734 | - | - | - | 0.3911 | 0.1823 | - |
|  | 17 | 1.4721 | 0.5384 | - | - | - | 0.1376 | 0.4008 | - |
|  | 18 | 1.6234 | 1.0162 | - | - | - | 0.2936 | 0.5226 | - |

Abbreviations: same as Table S4.

**Table S7** Sample sequencing data quality control and assembly results

| **Sample** | **Clean data base (bp)** | **Number of Reads** | **GC (%)** | **Q30 (%)** | **N50 (bp)** | **Contig Num.** | **Mapped (%)** |
| --- | --- | --- | --- | --- | --- | --- | --- |
| 0 mg | 13212325965 | 86020728 | 48.82 | 95.66 | 895 | 1055321 | 87.46 |
| 75 mg | 13944070998 | 90746616 | 49.35 | 95.62 | 964 | 1125326 | 87.27 |
| 150 mg | 13893148003 | 90266395 | 49.89 | 95.46 | 958 | 1183666 | 86.45 |

**Table S8** Ruminal volatile fatty acid profiles and pH in sheep exposed to PS-MPs

| **Items** | **Groups** | | | **SEM** | ***P*-value** |
| --- | --- | --- | --- | --- | --- |
|  | **0 mg/kg** | **75 mg/kg** | **150 mg/kg** |  |  |
| Acetate (A), mmol/L | 63.21 | 62.14 | 60.80 | 1.60 | 0.347 |
| Propionate (P), mmol/L | 18.42 | 19.12 | 18.55 | 0.58 | 0.229 |
| Isobutyrate, mmol/L | 0.80 | 0.89 | 0.78 | 0.09 | 0.461 |
| Isovalerate, mmol/L | 1.47 | 1.53 | 1.24 | 0.27 | 0.543 |
| Valerate, mmol/L | 0.56 | 0.53 | 0.62 | 0.05 | 0.266 |
| A:P | 3.43 | 3.26 | 3.36 | 0.11 | 0.349 |
| Total, mmol/L | 92.98^a^ | 89.76^b^ | 89.24^b^ | 1.95 | 0.003 |
| pH | 6.68 | 6.56 | 6.69 | 0.12 | 0.543 |

Note: Data are presented as mean ± SEM. Different lowercase letters indicate statistically significant differences among groups (*P* < 0.05).

**Table S9** Natural recovery of ruminal volatile fatty acids and pH after cessation of PS-MPs exposure in sheep

| **Items** | **Groups** | | | **SEM** | **P-value** |
| --- | --- | --- | --- | --- | --- |
|  | **Con** | **NR-75** | **NR-150** |  |  |
| Acetate (A), mmol/L | 65.38^c^ | 71.59^b^ | 78.62^a^ | 1.21 | < 0.001 |
| Propionate (P), mmol/L | 20.96 | 21.15 | 20.58 | 0.29 | 0.161 |
| Isobutyrate, mmol/L | 0.85 | 0.84 | 0.89 | 0.03 | 0.224 |
| Isovalerate, mmol/L | 1.47 | 1.53 | 1.56 | 0.06 | 0.334 |
| Valerate, mmol/L | 0.65 | 0.68 | 0.65 | 0.02 | 0.168 |
| A:P | 3.12^c^ | 3.39^b^ | 3.85^a^ | 0.08 | < 0.001 |
| Total, mmol/L | 97.90^b^ | 99.99^b^ | 104.46^a^ | 1.36 | 0.001 |
| pH | 6.85 | 6.68 | 6.53 | 0.21 | 0.334 |

Note: Data are presented as mean ± SEM. Different lowercase letters indicate statistically significant differences among groups (*P* < 0.05).

**Table S10** Changes in ruminal volatile fatty acids and pH in sheep supplemented with sodium butyrate after cessation of exposure

| Items | Groups | | | SEM | *P*-value |
| --- | --- | --- | --- | --- | --- |
|  | Ctrl | Ctrl+NaB | 150+NaB |  |  |
| Acetate (A), mmol/L | 70.46 | 74.57 | 72.85 | 2.19 | 0.203 |
| Propionate (P), mmol/L | 22.77 | 23.80 | 22.88 | 1.28 | 0.683 |
| Isobutyrate, mmol/L | 0.94 | 0.88 | 0.88 | 0.08 | 0.717 |
| Isovalerate, mmol/L | 1.73 | 1.71 | 1.63 | 0.16 | 0.795 |
| Valerate, mmol/L | 0.82 | 0.90 | 0.89 | 0.06 | 0.407 |
| A:P | 3.10 | 3.17 | 3.19 | 0.14 | 0.806 |
| Total, mmol/L | 107.16 | 113.38 | 110.07 | 4.03 | 0.331 |
| pH | 6.85 | 6.73 | 6.69 | 0.49 | 0.946 |

Note: Data are presented as mean ± SEM. Different lowercase letters indicate statistically significant differences among groups (*P* < 0.05).
